# Heavy Disease Burden of High Systolic Blood Pressure During 1990-2019: Highlighting Regional, Sex, and Age Specific Strategies in Blood Pressure Control

**DOI:** 10.3389/fcvm.2021.754778

**Published:** 2021-12-16

**Authors:** Ming-Ming Chen, Xingyuan Zhang, Ye-Mao Liu, Ze Chen, Haomiao Li, Fang Lei, Juan-Juan Qin, Yanxiao Ji, Peng Zhang, Jingjing Cai, Zhi-Gang She, Xiao-Jing Zhang, Zhibing Lu, Hui Liu, Hongliang Li

**Affiliations:** ^1^Department of Cardiology, Renmin Hospital, School of Basic Medical Science, Wuhan University, Wuhan, China; ^2^Institute of Model Animal, Wuhan University, Wuhan, China; ^3^Department of Cardiology, Zhongnan Hospital of Wuhan University, Wuhan, China; ^4^Medical Science Research Center, Zhongnan Hospital of Wuhan University, Wuhan, China; ^5^Department of Cardiology, The Third Xiangya Hospital, Central South University, Changsha, China; ^6^Department of Gastroenterology, Tongren Hospital of Wuhan University and Wuhan Third Hospital, Wuhan, China

**Keywords:** high systolic blood pressure, sociodemographic index regions, cardiovascular diseases, chronic kidney disease, disease burden

## Abstract

**Objective:** High systolic blood pressure (HSBP) remains the leading risk factor for mortality worldwide; however, limited data have revealed all-cause and cause-specific burdens attributed to HSBP at global and regional levels. This study aimed to estimate the global burden and priority diseases attributable to HSBP by region, sex, and age.

**Methods:** Based on data and evaluation methods from the Global Burden of Diseases, Injuries, and Risk Factors Study 2019, we estimated trends of age-standardized mortality rate (ASMR), the age-standardized rate of disability-adjusted life years (ASDRs), and the age-standardized rate of years lived with disability (ASYRs) attributable to HSBP during 1990-2019. Further, we analyzed cause-specific burdens attributable to HSBP by sex, age, year, and region.

**Results:** Globally, a significant downtrend was found in the ASMR attributed to HSBP while ASYRs did not change substantially during 1990-2019. The majority of HSBP burden has shifted from high-middle sociodemographic index (SDI) regions to lower SDI regions. All-cause and most cause-specific burdens related to HSBP were improved in high SDI regions but the downtrends have stagnated in recent years. Although many cause-specific deaths associated with HSBP declined, chronic kidney disease (CKD) and endocarditis associated deaths were aggravated globally and ischemic heart disease (IHD), atrial fibrillation and flutter, aortic aneurysm (AA), and peripheral artery disease (PAD) associated deaths were on the rise in low/low-middle/middle SDI regions. Additionally, males had higher disease burdens than females. Middle-aged people with CVDs composed the major subgroup affected by HSBP while older people had the highest ASMRs associated with HSBP.

**Conclusions:** This study revealed the global burden and priority diseases attributable to HSBP with wide variation by region, sex, and age, calling for effective and targeted strategies to reduce the prevalence and mortality of HSBP, especially in low/low-middle/middle SDI regions.

## Introduction

High systolic blood pressure (HSBP) represents a major health problem and is responsible for a dramatic economic burden worldwide (1, 2), affecting 4.06 billion people and leading to 10.8 million deaths in 2019 (3). Diseases causally associated with HSBP are devastating, especially cardiovascular diseases (CVDs), which are the leading contributor to mortality and disability (4). The growing evidence for the tight association of HSBP with CVDs, chronic kidney disease (CKD), coronavirus disease 2019, and other metabolic diseases has emphasized the importance of controlling systolic blood pressure (SBP) for preventing related complications (5–11). Globally, lifestyle and behavior interventions, combined with antihypertensive treatment, have been widely used to lower SBP, requiring a large amount of economic input (12–14). However, global SBP levels have remained stagnant or decreased marginally over the past four decades (12, 15). The reduction in overall mortality is another major concern in the context of SBP control but is not clearly defined at the global level. In addition, cause-specific burdens attributed to HSBP have rarely been summarized worldwide, while fragmented research has masked the overall picture of HSBP. Thus, there is an urgent need for an up-to-date analysis of the disease burdens attributable to HSBP globally and regionally, guiding targeted prevention and control strategies in different regions.

This analysis thoroughly explores the temporal trend of disease burdens associated with HSBP from 1990 to 2019 using data from the Global Burden of Disease, Injuries, and Risk Factors Study (GBD) 2019 and describes the sex disparities, age differences, and regional patterns of disease burdens associated with HSBP in detail. The overview of global and regional disease burdens associated with HSBP provides an essential guide for implementing health policies to prevent HSBP mortality and decrease regional disparities.

## Methods

### Data Sources

GBD 2019 is a multinational collaborative research with a rule-based synthesis method used for data on the incidence, prevalence, and death associated with diseases and injuries for each country worldwide (2). Based on GBD 2019, data on deaths, disability-adjusted life years (DALYs), years of life lost (YLLs) and years of life lived with disability (YLDs) attributable to HSBP by sex (female, male, and both), age (5-year groups within the ages of 20-95 years, <20, and ≥95 years), year (1990-2019), and location were available through the GBD Results Tool (http://ghdx.healthdata.org/gbd-results-tool). The 204 countries and territories in GBD 2019 were grouped into 21 regions according to geography and sociodemographic index (SDI) (high, high-middle, middle, low-middle, and low SDI region), which is a summary measure of overall development, based on educational attainment, fertility, and income per capita within a location. Data sources, methodologies of GBD 2019, and comparative risk assessment specifically for HSBP have been presented in detail in previous researches (2).

### Definitions

HSBP is defined by GBD 2019 according to a theoretical minimum risk exposure level (TMREL) of ≥110-115 mm Hg, which is the level of exposure that minimizes risk at the population level (2). Detailed information about the process of data selection and data inputs has been published previously (3).

We used age-standardized mortality rates (ASMRs), age-standardized rates of DALYs (ASDRs), and age-standardized rates of YLDs (ASYRs) to quantify the HSBP-related burden. Deaths were regarded as the number of deaths that occurred in a population over a given period. Mortality data was traced to vital registration data coded in the International Classification of Disease system or household mortality surveys. DALYs were used to estimate the global disease burden of specific causes, combined with the burden caused by YLLs (multiplying observed deaths among individuals of a specific age in the year of interest by the age-specific reference life expectancy estimated using life table methods) and YLDs (years lived with any short-term or long-term health loss weighted for severity by the disability weights).

In GBD 2019, all causes were classified into four levels (3). In particular, non-communicable diseases are level 1, including 12 diseases for the level 2 groupings, such as cardiovascular diseases. Furthermore, level 3 represented more detailed causes within the level 2 categories, such as stroke within cardiovascular diseases, while level 4 included subcauses of some level 3 causes, such as ischemic stroke within the stroke. In GBD 2019, HSBP was found to be associated with 2 levels 2 causes of death and DALYs (cardiovascular diseases, diabetes, and kidney diseases) for both sexes. The number of causes of death and DALYs attributable to HSBP was 12 for level 3 and 10 for level 4 causes for both sexes.

### Attributable Burden Estimation

The estimation methods applied in this study have been described in detail elsewhere (1–3). In our research, we used the ASMRs, ASDRs, and ASYRs with 95% uncertainty intervals (UIs) to quantify the burden of disease attributed to HSBP by age, sex, year, and location. The cause of death ensemble model was used to predict mortality based on available data and covariates (16). UIs were defined by 1,000 draw-level estimates for each parameter, and 95% UIs were the 25th and 75th values of the ordered 1,000 estimates. The attributable proportions of ASMRs compared by age, sex, year, and location were evaluated by population attributable fractions (PAFs), which presented the ASMRs that could decrease if the exposure to HSBP was eliminated to an alternative ideal situation. The equation of PAF for SBP is defined as follows (2, 17): $PAF_{oasct}=\frac{\int_{x}^{u}=1_{os}^{RR}\left(x\right)P_{asct}\left(x\right)dx-RR_{oas}\left(TMREL\right)}{\int_{x=1}^{u}RR_{oas}\left(x\right)P_{asct}\left(x\right)dx}$, where RR_oas_(x) is the relative risk as a function of exposure level (x) for SBP, cause (o), age group (a), and sex (s). P_asct_ (x) is the distribution of exposure of SBP according to age group (a), sex (s), country (c), and year (t). The lowest level of observed exposure (l) and the highest level of observed exposure (u) are described in the denominator. Furthermore, we analyzed the percent change in PAFs of ASMRs related to HSBP from 1990 to 2019.

### Statistical Analysis

We calculated the estimated annual percentage change (EAPC) of ASMRs, ASDRs, and ASYRs to reflect their change trends from 1990 to 2019 (18). Age-standardized rates (ASRs) (per 100,000 population) were calculated by the following formula: $ASR=\frac{\overset{A}{\underset{i=1}{\sum }}aiwi}{\overset{A}{\underset{i=1}{\sum }}wi}\times 100000$, where *ai* represents the specific age ratio, and *wi* represents the number of persons (or weight). The EAPC was calculated based on the formula 100 × (exp(β) – 1), and the 95% confidence interval (CI) was obtained from the linear regression model. It is assumed that the natural logarithm of ASR is linear over time; thus, *Y* = α + β*X* + ε, where *Y* = ln (ASR)*, X* = calendar year, and ε = the error term. If the EAPC and the lower boundary of its 95% CI were both positive, the ASR was deemed to have an increasing trend. Conversely, if the EAPC estimation and the upper boundary were negative, the ASMR was considered to have a decreasing trend.

Additionally, we analyzed the association between SDI and disease burden attributable to HSBP by location and year using a Gaussian process regression with a Loess smoother on SDI to estimate the relationship. The analytical methods used have been published previously (19), and the related codes can be accessed at http://ghdx.healthdata.org/gbd-2019/code. All statistics were performed using the R program (Version 4.0.4, R core team). A *p*-value of <0.05 was considered statistically significant.

## Results

### Impact of HSBP on Global Disease Burden

Despite declining trends of ASMRs and ASDRs, ASYRs remained unchanged over the study period. From 1990 to 2019, the ASMR attributed to HSBP declined from 197.87(95% UI: 174.93, 220.93) to 138.88(95% UI: 121.25, 155.73) for both sexes with an EPAC of −1.32 (95% CI: −1.36, −1.27) (Table 1). The ASDRs attributable to HSBP have declined from 3953.92 (95% UI: 3557.53, 4359.13) to 2885.57 (95% UI: 2580.75, 3201.05) for both sexes with an EPAC of −1.17 (95% CI: −1.22, −1.13) (Supplementary Table 1). However, ASYRs caused by HSBP remained stable from 256.03 (95% UI: 184.76, 330.07) in 1990 to 258.54 (95% UI: 185.85, 331.93) in 2019 with an EAPC of 0.09 (95% CI: 0.06, 0.11) (Table 1).

**Table 1:** ASMRs and ASYRs attributable to HSBP by sex, SDI, GBD regions in 1990, 2019 and EAPC from 1990 to 2019.

	**1990**	**2019**	**1990-2019**	**1990**	**2019**	**1990-2019**
**Characteristics**	**ASMR per 100,000 (95% UI)**	**ASMR per 100,000 (95% UI)**	**EAPC (95% CI)**	**ASYRs per 100,000 (95% UI)**	**ASYRs per 100,000 (95% UI)**	**EAPC (95% CI)**
**Global**						
Overall	197.87 (174.93,220.93)	138.88 (121.25,155.73)	−1.32 (−1.36, −1.27)	256.03 (184.76,330.07)	258.54 (185.85,331.93)	0.09 (0.06,0.11)
Male	218.54 (192.84,243.49)	160.13 (138.91,180.79)	−1.11 (−1.14, −1.08)	246.07 (176.00,318.97)	254.06 (183.05,330.16)	0.21 (0.18,0.24)
Female	178.86 (154.91,201.06)	119.66 (102.33,136.86)	−1.53 (−1.59, −1.47)	263.40 (188.91,339.86)	261.61 (187.94,336.11)	−0.01 (−0.04,0.02)
**SDI**						
High SDI	160.45 (138.52,180.19)	69.76 (58.67,79.66)	−3.24 (−3.44, −3.03)	262.57 (189.47,338.55)	209.39 (150.28,270.86)	−0.89 (−0.98, −0.79)
High-middle SDI	234.74 (205.16,262.04)	147.83 (126.74,168.89)	−1.85 (−2.04, −1.67)	279.78 (201.44,362.42)	275.11 (198.93,354.98)	0.02 (0.00,0.04)
Middle SDI	200.27 (175.72,226.27)	168.54 (147.10,190.77)	−0.44 (−0.51, −0.37)	257.97 (184.58,337.52)	300.25 (215.76,387.30)	0.66 (0.62,0.69)
Low-middle SDI	184.08 (161.13,208.66)	166.81 (144.97,189.46)	−0.34 (−0.40, −0.29)	197.38 (142.31,256.68)	226.05 (163.12,291.12)	0.54 (0.52,0.57)
Low SDI	188.88 (162.30,218.75)	169.85 (147.99,191.20)	−0.35 (−0.44, −0.27)	188.58 (138.06,244.25)	208.56 (151.69,269.10)	0.44 (0.41,0.48)

*ASMR, age-standardized mortality rate; ASYRs, age-standardized rate of years lived with disability; HSBP, high systolic blood pressure; EAPC, estimated annual percentage change; UI, uncertainty interval; CI, confidence interval; SDI, sociodemographic index*.

Males had higher burdens related to HSBP than females (Table 1). The ASMRs attributable to HSBP declined from 218.54 (95% UI: 192.84, 243.49) to 160.13(95% UI: 138.91, 180.79) for males and from 178.86 (95% UI: 154.91, 201.06) to 119.66 (95% UI: 102.33, 136.86) for females during 1990-2019. The ASDRs caused by HSBP declined from 4538.11 (95% UI: 4060.32, 5008.08) to 3448.86 (95% UI: 3060.06, 3837.69) for males and from 3403.35 (95% UI: 3025.28, 3766.52) to 2354.72 (95% UI: 2075.57, 2634.68) for females, from 1990 to 2019 (Supplementary Table 1). Both ASMR and ASDR were lower in females than in males in both 1990 and 2019. Notably, ASYRs attributable to HSBP was lower in males than in females in both 1990 and 2019. The EAPC of the ASYR attributable to HSBP was 0.21 (95% CI: 0.18, 0.24) for males and −0.01 (95% CI: −0.04, 0.02) for females.

ASMRs and ASYRs varied widely across age groups (Supplementary Figure 1). Older people had the highest ASMRs imposed by HSBP during the study period. The ASMRs in the <20-year age groups were the lowest. From 1990 to 2019, the 70-74, 75-79, 80-84, 85-89, 90-94, and 95 plus year age groups exhibited a slight downtrend in ASMRs attributed to HSBP. Similarly, the ASYR in older people was higher than that in young people, and the <20, 20-24 age groups were the lowest.

### Impact of HSBP in Different Regions

The all-cause burden of HSBP was alleviated significantly in high/high-middle SDI regions and is now still heavy in low/low-middle/middle SDI regions (Table 1). In 2019, the ASMR attributable to HSBP was lower in high (69.76, 95% UI: 58.67, 79.66) and high-middle SDI regions (147.83, 95% UI: 126.74, 168.89) than those in middle (168.54, 95% UI: 147.10, 190.77), low-middle (166.81, 95% UI: 144.97, 189.46), and low SDI regions (169.85, 95% UI: 147.99, 191.20). From 1990 to 2019, the EAPCs of ASMR attributable to HSBP were lower in the high (−3.24, 95% CI: −3.44, −3.03) and high-middle SDI regions (−1.85, 95% CI: −2.04, −1.67) than in the middle (−0.44, 95% CI: −0.51, −0.37), low-middle (−0.34, 95% CI: −0.40, −0.29), and low SDI regions (−0.35, 95% CI: −0.44, −0.27). Similar patterns were observed for ASDRs (Supplementary Table 1). ASYR attributable to HSBP was decreased slightly in high SDI regions with the EAPC of −0.89 (95% CI: −0.98, −0.79) while that of other regions was increased with the EAPC of 0.02 (95% CI: 0.00, 0.04) in the high-middle, 0.66 (95% CI: 0.62, 0.69) in middle, 0.54 (95% UI: 0.52, 0.57) in low-middle, and 0.44 (95% CI: 0.41, 0.48) in low SDI regions.

**Figure 1:**
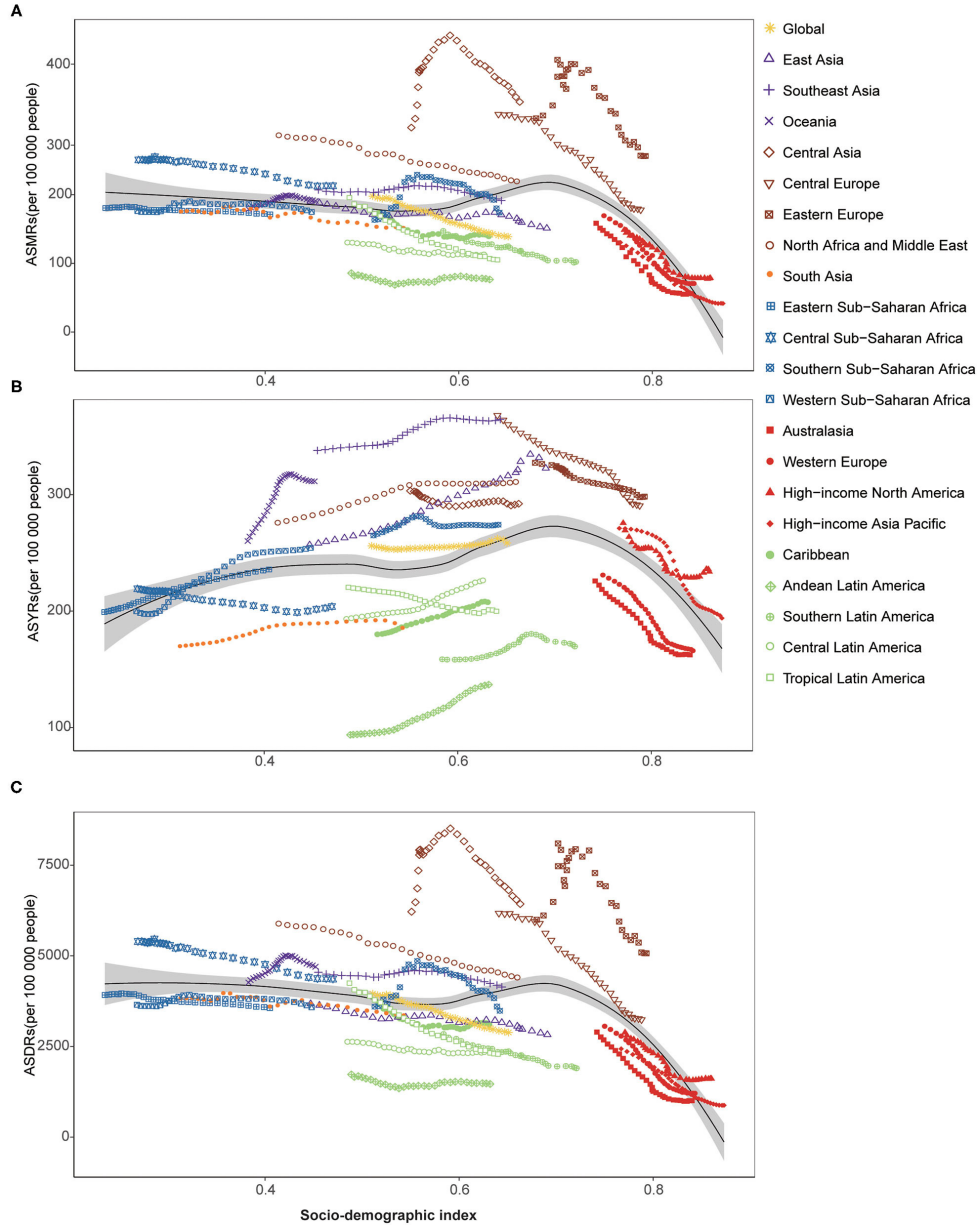
ASMRs, ASYRs, and ASDRs attributable to HSBP across 21 GBD regions by SDI for both sexes combined, 1990-2019. **(A)** ASMRs attributable to HSBP across 21 GBD regions by SDI for both sexes combined, 1990-2019; **(B)** ASYRs attributable to HSBP across 21 GBD regions by SDI for both sexes combined, 1990-2019. **(C)** ASDRs attributable to HSBP across 21 GBD regions by SDI for both sexes combined, 1990-2019. ASMRs, age-standardized mortality rates; ASYRs, age-standardized rate of years lived with disability; ASDR, age-standardized disability-adjusted life years; HSBP, high systolic blood pressure; GBD, global burden of disease, injuries, and risk factors study; SDI, sociodemographic index.

Further analyses of the associations between SDI and ASMRs, ASYRs, and ASDRs illustrated the above findings again (Figure 1). The estimated relation between SDI and expected ASMR is generally negative, with a steepening downtrend after SDI of approximately 0.68. The uptrend between 0.6 and 0.68 was mainly affected by Central Asia and Eastern Europe. Importantly, all four regions with the highest SDI experienced a steep decline in ASMRs attributed to HSBP during the study period (Figure 1A). The pattern of ASDR was similar (Figure 1C). In contrast, the estimated relation between SDI and the expected ASYRs is generally active until the SDI is approximately 0.7 (Figure 1B). All four regions with the highest SDI presented downtrends during the study period.

Geographically, all-cause burdens of HSBP were generally lower in GBD regions with high SDI than in those with low SDI. From 1990 to 2019, ASMRs declined in 17 GBD regions with the 4 lowest EAPCs of ASMRs attributed to HSBP from 1990 to 2019 in four regions with the highest SDI, including High-income Asia Pacific, Australasia, Western Europe, and High-income North America (Supplementary Table 2). In 2019, High-income Asia Pacific (41.90, 95% UI: 33.84, 48.79), High-income North America (78.66, 95% UI: 65.78, 90.81), Western Europe (70.67, 95% UI: 59.21, 80.20), and Australasia (56.22, 95% UI: 45.36, 66.93) maintained low levels of ASMRs imposed by HSBP. Similar phenomena were observed in ASDRs and ASYRs associated with HSBP in these 4 regions (Supplementary Tables 2, 3). In contrast, regions with relatively low SDIs had high ASMRs, ASDR, and ASYRs. Interestingly, the highest ASMR (335.07, 95% UI: 282.32, 382.09) and ASDR (6429.35, 95% UI: 5614.52, 7222.91) attributed to HSBP were found in Central Asia in 2019, while the highest HSBP-related ASYR was observed in Southeast Asia (364.61, 95% UI: 263.39, 466.83), both of which are middle-SDI regions (Supplementary Table 2).

**Figure 2:**
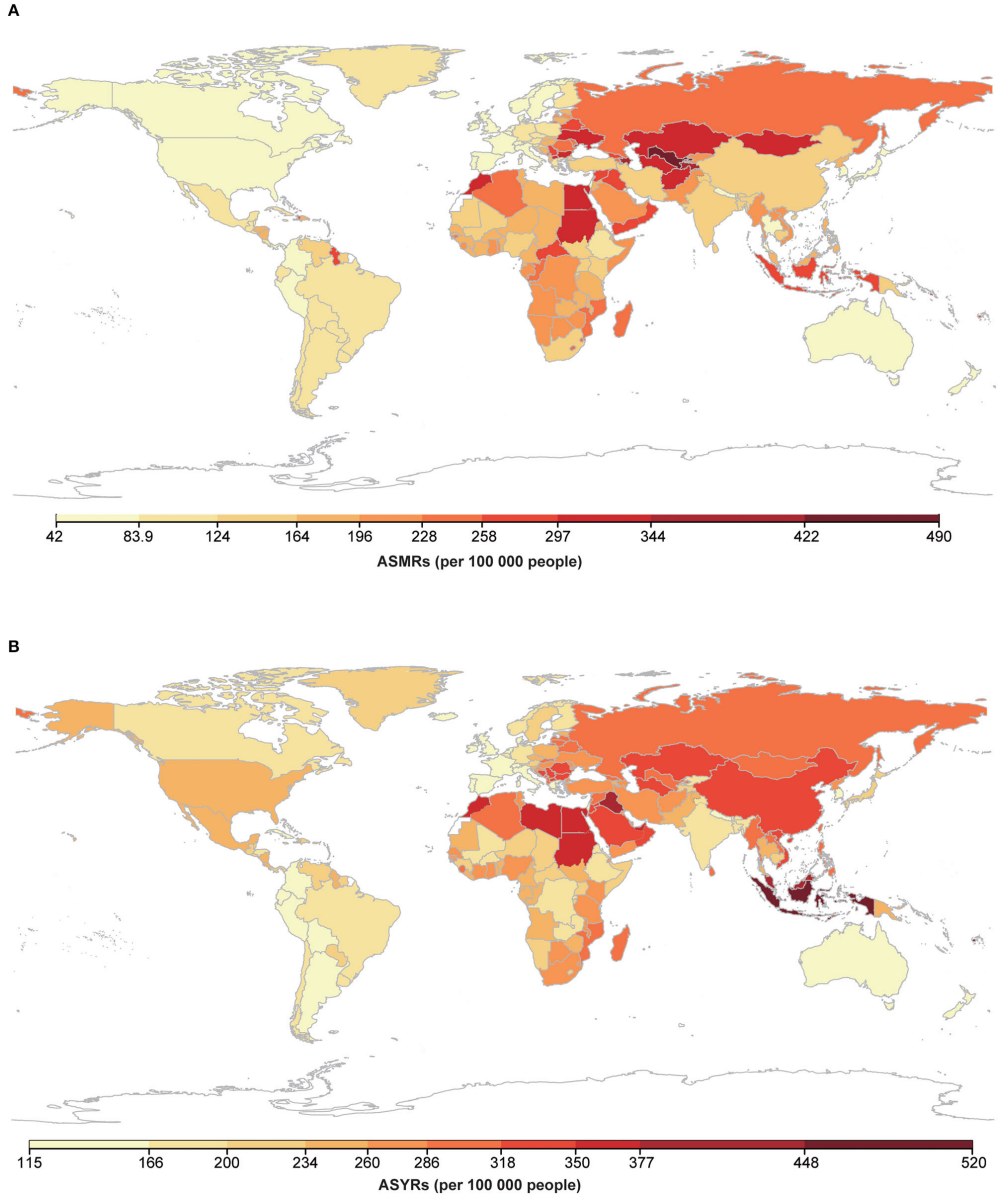
Map of ASMRs and ASYRs attributable to HSBP for both sexes in 2019. **(A)** Map of ASMRs attributable to HSBP for both sexes in 2019; **(B)** Map of ASYRs attributable to HSBP for both sexes in 2019. Abbreviations as in Figure 1.

There were also dramatic differences in country-level ASMRs and the ASYRs (Figure 2, Supplementary Figure 2 and Supplementary Table 4). Generally, high ASMRs and ASYRs were mainly distributed in Asian and African countries, while countries in North America, South America, and Oceania had relatively low levels. The highest ASMR attributable to HSBP was observed in Uzbekistan and the lowest was observed in Japan in 2019. The highest HSBP-related ASYR was observed in Nauru and the lowest was observed in Bolivia. In particular, some developed countries, such as the Republic of Korea, United Kingdom, United States of America, and Australia, have experienced a sharp decrease from 1990 to 2019 (Supplementary Table 4). In contrast, ASMRs in many developing countries, such as China, Zimbabwe, Mozambique, and Egypt, increased significantly or remained unimproved from 1990 to 2019.

### Impact of HSBP on Different Diseases

Overall, HSBP is the leading risk factor attributed to CVDs and CKD mortality worldwide, and the global burdens of CVDs attributable to HSBP have improved slightly while CKD has worsened (Table 2). There were two level 2 causes of HSBP-related ASMRs in 2019, of which CVDs were the leading cause of ASMRs due to HSBP (127.54, 95% UI: 110.59, 143.80) followed by diabetes and kidney diseases (11.34, 95% UI: 9.88, 12.68). Of CVD ASMRs worldwide in 2019, 53.18% (95% UI: 47.27, 59.01) were attributable to HSBP while the corresponding proportion was 29.91% (95% UI: 26.77, 32.67) for diabetes and kidney diseases. The first three age-standardized PAFs of level 3 causes of ASMRs in CVDs attributable to HSBP were 100.00% (95% UI: 100.00, 100.00) for hypertensive heart disease (HHD), 52.78% (95% UI: 42.58, 62.58) for IHD and 51.79% (95% UI: 43.64, 59.64) for stroke. The first three level 3 causes of ASMRs attributable to HSBP among CVDs were IHD (62.26, 95% UI: 49.90, 75.51), stroke (43.60, 95% UI: 36.19, 51.06), and HHD (15.16, 95% UI: 11.20, 16.75), and they together accounted for 94.89% of all HSBP-related ASMRs among CVDs. Notably, in addition to endocarditis, ASMRs of other CVDs attributable to HSBP all decreased from 1990 to 2019, with the lowest EAPC in subarachnoid hemorrhage (−3.19, 95% CI: −3.47, −2.90) and the highest in endocarditis (0.73, 95% CI: 0.42, 1.04). The only GBD level 3 cause in diabetes and kidney diseases attributable to HSBP was CKD with an ASMR of 11.34 (95% UI: 9.88, 12.68) and an age-standardized PAF of 61.98% (95% UI: 55.75, 67.41). Unfortunately, the EAPC in ASMRs of CKD due to HSBP was 0.70 (95% CI: 0.60, 0.80).

**Table 2:** Global ASMRs and ASDRs attributable to HSBP for both sexes combined of each disease in 2019, EAPC and percentage change from 1990 to 2019.

	**ASMRs**				**ASDRs**			
**Characteristics**	**ASMR, per 100,000, 2019 (95% UI)**	**EAPC, 1990-2019 (95% CI)**	**Age-standardized PAF%, 2019 (95% UI)**	**Percentage change in age-standardized PAF, 1990-2019 (95% UI)**	**ASDR, per 100000, 2019 (95 % UI)**	**EAPC, 1990-2019 (95% CI)**	**Age-standardized PAF%, 2019 (95% UI)**	**Percentage change in age-standardized PAF, 1990-2019 (95% UI)**
**Cardiovascular diseases**	127.54 (110.59,143.80)	−1.45 (−1.50, −1.41)	53.18 (47.27,59.01)	0.00 (−0.03,0.03)	2621.16 (2339.38,2920.64)	−1.32 (−1.37, −1.27)	53.89 (48.96,58.37)	0.03 (0.00,0.05)
Hypertensive heart disease	15.16 (11.20,16.75)	−0.74 (−0.91, −0.57)	100.00 (100.00,100.00)	0.00(0.00,0.00)	268.19 (204.57,298.07)	−1.02 (−1.18, −0.86)	100.00 (100.00,100.00)	0.00 (0.00,0.00)
Ischemic heart disease	62.26 (49.90,75.51)	−1.47 (−1.52, −1.43)	52.78 (42.58,62.58)	−0.03 (−0.05,0.00)	1217.90 (1032.03,1412.44)	−1.27 (−1.32, −1.23)	54.28 (46.17,61.67)	0.00 (−0.02,0.02)
Stroke	43.60 (36.19,51.06)	−1.64 (−1.77, −1.51)	51.79 (43.64,59.64)	0.02 (−0.02,0.07)	969.4 (823.77,1110.20)	−1.46 (−1.57, −1.34)	54.83 (47.48,61.48)	0.05 (0.01,0.10)
Intracerebral hemorrhage	20.47 (16.52,24.49)	−1.33 (−1.56, −1.10)	56.82 (46.37,66.86)	0.07 (0.02,0.13)	489.23 (406.00,573.54)	−1.31 (−1.51, −1.11)	58.75 (49.37,67.11)	0.10 (0.05,0.15)
Subarachnoid hemorrhage	2.61 (2.05,3.13)	−3.19 (−3.47, −2.90)	55.95 (46.24,65.27)	0.09 (0.02,0.16)	76.43 (61.04,91.63)	−2.79 (−3.03, −2.55)	56.00 (47.36,64.36)	0.11 (0.05,0.18)
Ischemic stroke	20.53 (15.41,26.10)	−1.72 (−1.81, −1.62)	47.19 (36.01,59.05)	−0.02 (−0.06,0.02)	403.74 (318.26,487.77)	−1.34 (−1.43, −1.26)	50.54 (40.30,59.99)	0.00 (−0.04,0.05)
Aortic aneurysm	0.76 (0.58,0.92)	−1.43 (−1.57, −1.30)	34.11 (26.81,41.09)	−0.09 (−0.12, −0.07)	15.12 (12.33,17.89)	−1.31 (−1.43, −1.19)	36.93 (30.63,43.17)	−0.06 (−0.09, −0.03)
Other cardiovascular and circulatory diseases	1.22 (1.04,1.43)	−1.61 (−1.68, −1.54)	34.20 (29.98,38.84)	−0.05 (−0.08, −0.01)	34.70 (29.58,41.70)	−1.07 (−1.13, −1.02)	31.33 (27.91,35.33)	0.03 (−0.06,0.10)
Atrial fibrillation and flutter	1.46 (1.12,1.85)	−0.30 (−0.34, −0.26)	33.39 (26.55,40.69)	−0.07 (−0.11, −0.04)	41.89 (31.62,54.61)	−0.26 (−0.29, −0.23)	39.08 (33.87,44.53)	−0.05 (−0.09, −0.02)
Endocarditis	0.28 (0.19,0.38)	0.73 (0.42,1.04)	32.22 (24.99,40.21)	0.03 (−0.05,0.13)	6.68 (4.81,8.43)	0.46(0.25,0.67)	30.44 (24.55,36.63)	0.20 (0.01,0.42)
Rheumatic heart disease	0.95 (0.63,1.45)	−2.85 (−2.89, −2.81)	24.78 (16.78,39.08)	0.05 (−0.04,0.14)	29.72 (20.33,41.90)	−2.61 (−2.66, −2.56)	22.42 (15.54,31.82)	0.04 (−0.05,0.13)
Peripheral artery disease	0.25 (0.14,0.48)	−0.79 (−0.89, −0.70)	25.32 (18.08,34.05)	−0.12 (−0.16, −0.09)	5.34 (3.34,8.53)	−0.99 (−1.05, −0.92)	27.36 (21.09,34.07)	−0.09 (−0.13, −0.06)
Non-rheumatic valvular heart disease	0.54 (0.37,0.76)	−0.68 (−0.77, −0.59)	24.09 (16.88,33.35)	−0.06 (−0.14,0.01)	8.03 (6.07,10.21)	−0.99 (−1.09, −0.89)	22.37 (17.15,28.22)	−0.03 (−0.09,0.04)
Non-rheumatic calcific aortic valve disease	0.54 (0.37,0.76)	−0.68 (−0.77, −0.59)	30.83 (21.63,42.88)	−0.15 (−0.21, −0.09)	8.03 (6.07,10.21)	−0.99 (−1.09, −0.89)	33.62 (25.98,42.46)	−0.12 (−0.16, −0.06)
Cardiomyopathy and myocarditis	1.06 (0.78,1.37)	−2.03 (−2.14, −1.91)	23.98 (18.63,30.33)	−0.08 (−0.15,0.04)	24.19 (18.54,29.46)	−1.22 (−1.31, −1.12)	21.07 (17.24,25.09)	0.02 (−0.08,0.17)
Other cardiomyopathy	1.06 (0.78,1.37)	−2.03 (−2.14, −1.91)	33.96 (26.57,42.71)	−0.04 (−0.09,0.02)	24.19 (18.54,29.46)	−1.22 (−1.31, −1.12)	33.24 (27.56,39.46)	0.02 (−0.07,0.11)
**Diabetes and kidney diseases**	11.34 (9.88,12.68)	0.70 (0.60,0.80)	29.91 (26.77,32.67)	0.07 (0.02,0.10)	264.41 (230.09,298.96)	0.61 (0.52,0.70)	19.24 (16.49,22.09)	−0.01 (−0.06,0.03)
Chronic kidney disease	11.34 (9.88,12.68)	0.70 (0.60,0.80)	61.98 (55.75,67.41)	0.04 (0.02,0.06)	264.41 (230.09,298.96)	0.61 (0.52,0.70)	51.36 (45.96,56.42)	0.08 (0.04,0.11)
Chronic kidney disease due to hypertension	5.88 (4.95,6.82)	0.64 (0.57,0.72)	100.00 (100.00,100.00)	0.00 (0.00,0.00)	123.41 (106.86,142.66)	0.49 (0.42,0.56)	100.00 (100.00,100.00)	0.00 (0.00,0.00)
Chronic kidney disease due to diabetes mellitus type 2	2.45 (1.69,3.22)	1.05 (0.89,1.21)	47.50 (34.75,58.87)	0.04 (0.01,0.07)	53.99 (37.31,70.83)	0.97 (0.82,1.12)	44.91 (32.54,55.93)	0.06 (0.03,0.10)
Chronic kidney disease due to other and unspecified causes	1.69 (1.09,2.38)	0.58 (0.42,0.74)	42.76 (30.52,53.58)	0.06 (0.02,0.11)	47.96 (32.60,64.42)	0.59 (0.49,0.70)	32.77 (22.89,42.13)	0.15 (0.06,0.22)
Chronic kidney disease due to glomerulonephritis	0.93 (0.63,1.31)	0.39 (0.34,0.44)	40.25 (29.46,50.18)	0.07 (0.03,0.12)	25.79 (17.47,36.17)	0.50 (0.46,0.54)	29.88 (21.29,38.52)	0.13 (0.07,0.20)
Chronic kidney disease due to diabetes mellitus type 1	0.38 (0.22,0.60)	0.65 (0.52,0.79)	38.43 (27.65,48.99)	0.15 (0.11,0.21)	13.25 (7.81,20.41)	0.58 (0.46,0.70)	33.98 (24.03,44.15)	0.18 (0.12,0.25)

*ASMR, age-standardized rate of mortality rate; ASDR, age-standardized rate of disability-adjusted life years; HSBP, high systolic blood pressure; PAF, population attributable fraction; EAPC, estimated annual percentage change; UI, uncertainty interval; CI, confidence interval*.

**Figure 3:**
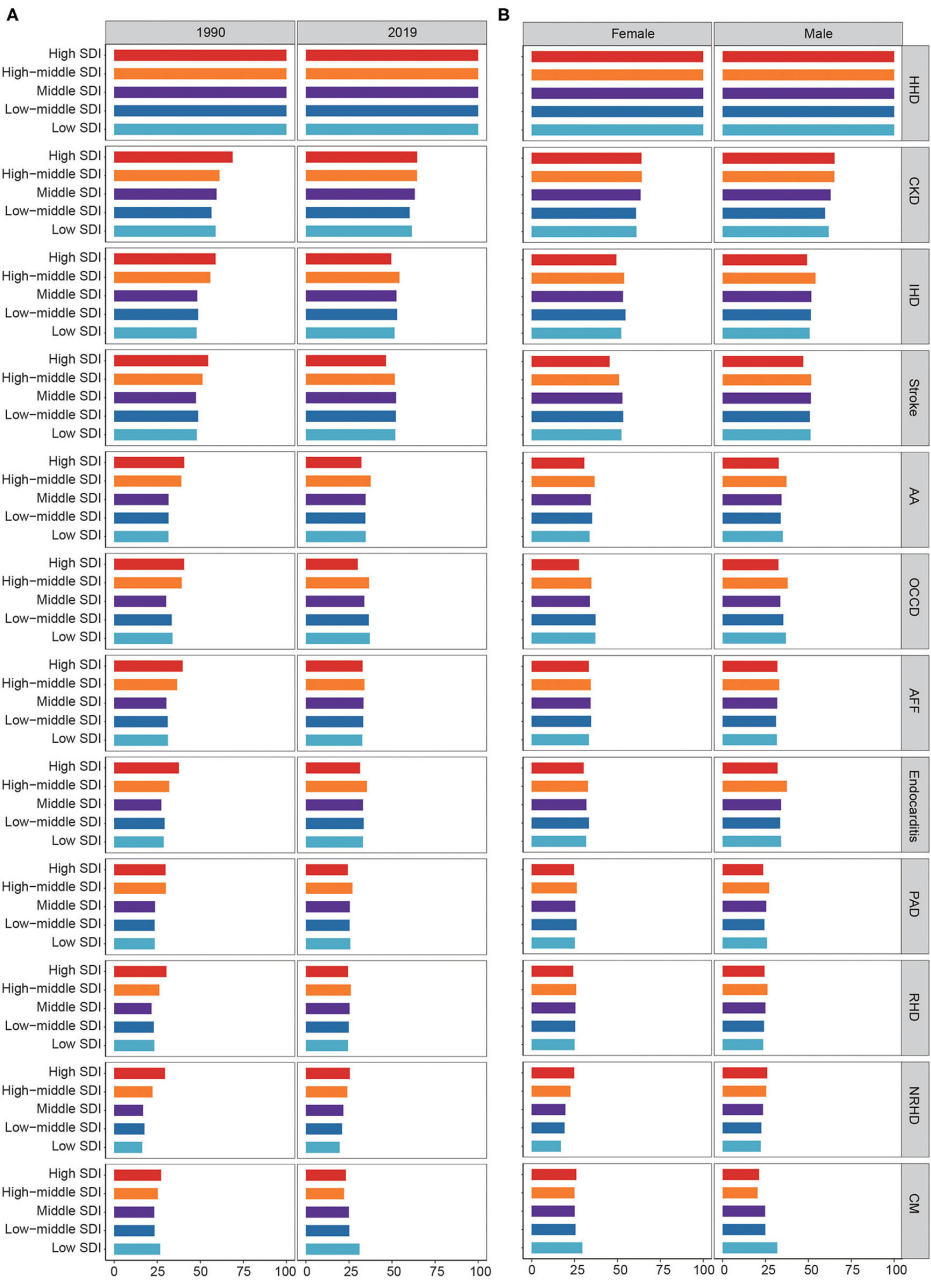
Fraction of disease ASMRs attributable to HSBP by SDI region. **(A)** Fraction of disease ASMRs attributable to HSBP by SDI region in 1990 and 2019. **(B)** Fraction of disease ASMRs attributable to HSBP by SDI region for female and male in 2019. ASMR, age-standardized mortality rate; HSBP, high systolic blood pressure; SDI, sociodemographic index; HHD, hypertensive heart disease; CKD, chronic kidney disease; IHD, ischemic heart disease; AA, aortic aneurysm; OCCD, other cardiovascular and circulatory diseases; AFF, atrial fibrillation and flutter; PAD, peripheral artery disease; RHD, rheumatic heart disease; NRHD, non-rheumatic valvular heart disease; CM, cardiomyopathy and myocarditis.

Age-standardized PAFs of diseases due to HSBP in high SDI regions decreased, while those in low/low-middle/middle SDI regions increased from 1990 to 2019 (Figure 3A). In 1990, the high SDI region had the highest PAFs of ASMRs associated with HSBP for most GBD level 3 causes. From 1990 to 2019, the attributable proportions of ASMRs related to HSBP in high SDI regions declined for every level 3 cause, except HHD. Nevertheless, the age-standardized PAFs of ASMRs due to HSBP for all level 3 causes increased in low/low-middle/middle SDI regions. In contrast, high-middle SDI regions experienced minimal changes. In 2019, the high SDI region had the lowest attributable proportions of ASMRs associated with HSBP for IHD, stroke, AA, other cardiovascular and circulatory diseases, endocarditis, PAD, and rheumatic heart disease (RHD). High-middle SDI region had the highest attributable proportions of ASMRs due to HSBP for IHD, AA, atrial fibrillation and flutter, endocarditis, PAD, and RHD in 2019. Similar phenomena in 2019 were observed in both males and females (Figure 3B).

**Figure 4:**
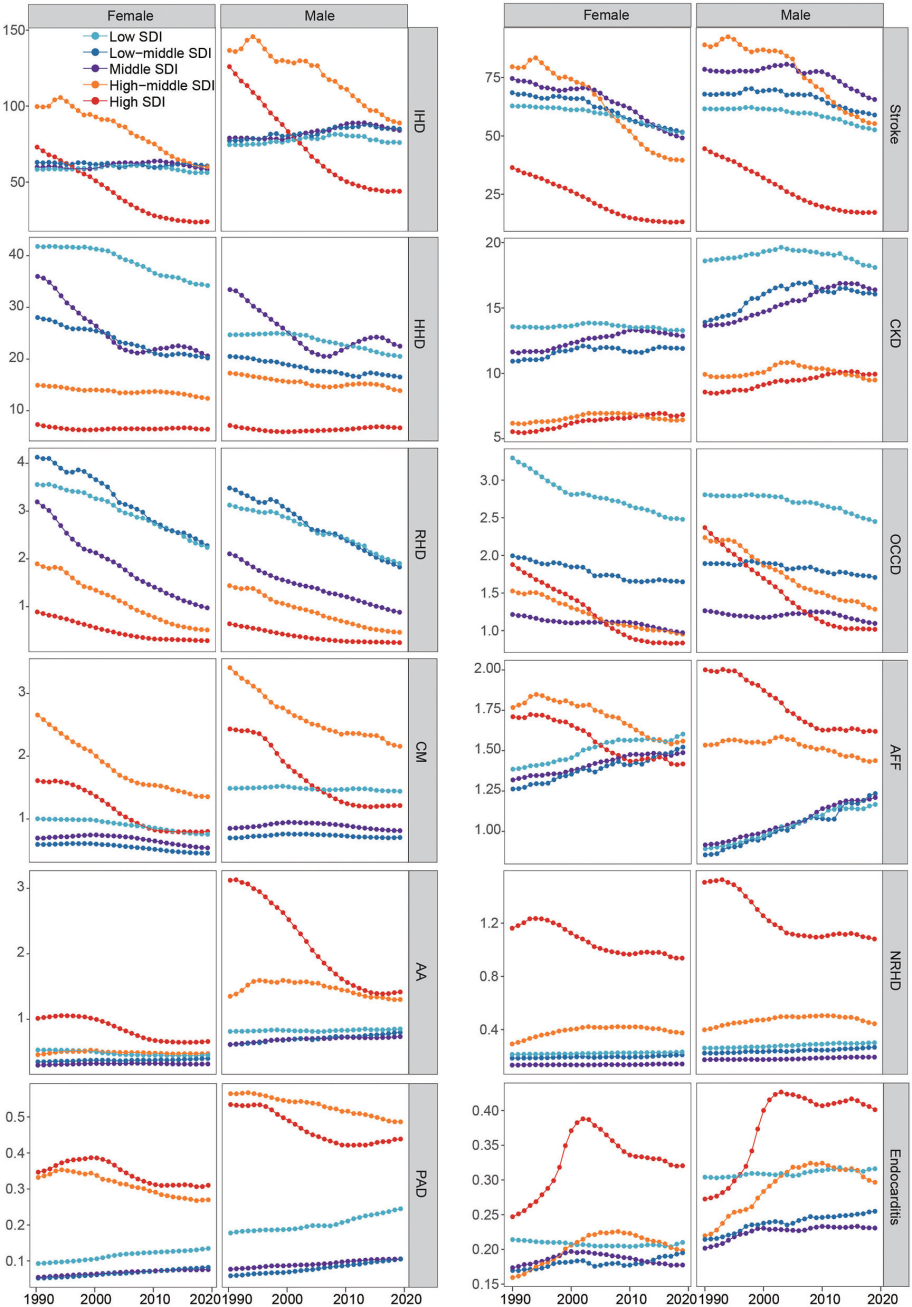
ASMRs of 12 causes attributable to HSBP across SDI regions for female and male, 1990-2019. Abbreviations as in Figure 3.

The ASMRs for most diseases due to HSBP in high/high-middle SDI regions declined dramatically, while there were slight changes in low/low-middle/middle SDI regions over the study period (Figure 4). Over the study period, ASMRs of IHD, stroke, HHD, RHD, other cardiovascular and circulatory diseases, cardiomyopathy and myocarditis, atrial fibrillation and flutter, and PAD decreased in high/high-middle regions. Although unobtrusive ASMR trends of AA and non-rheumatic valvular heart disease in the high-middle SDI region were observed, dramatic downtrends were observed in the high SDI regions. In contrast, low/low-middle/middle SDI regions experience inconspicuous improvements in ASMRs for most diseases. Despite improvements in high SDI regions, trends have flatten over the recent few years, and in 2019, ASMRs of AA, non-rheumatic valvular heart disease, and PAD in high SDI regions were still higher than those in low/low-middle/middle SDI regions. In particular, there were no substantial improvements in CKD or endocarditis in all SDI regions from 1990 to 2019. Furthermore, males had higher ASMRs for most diseases related to HSBP than females (Figure 4). Notably, the downtrends of ASMRs in IHD, atrial fibrillation and flutter, cardiomyopathy and myocarditis, AA, and non-rheumatic valvular heart disease in males were more significant than those in females across high SDI regions.

**Figure 5:**
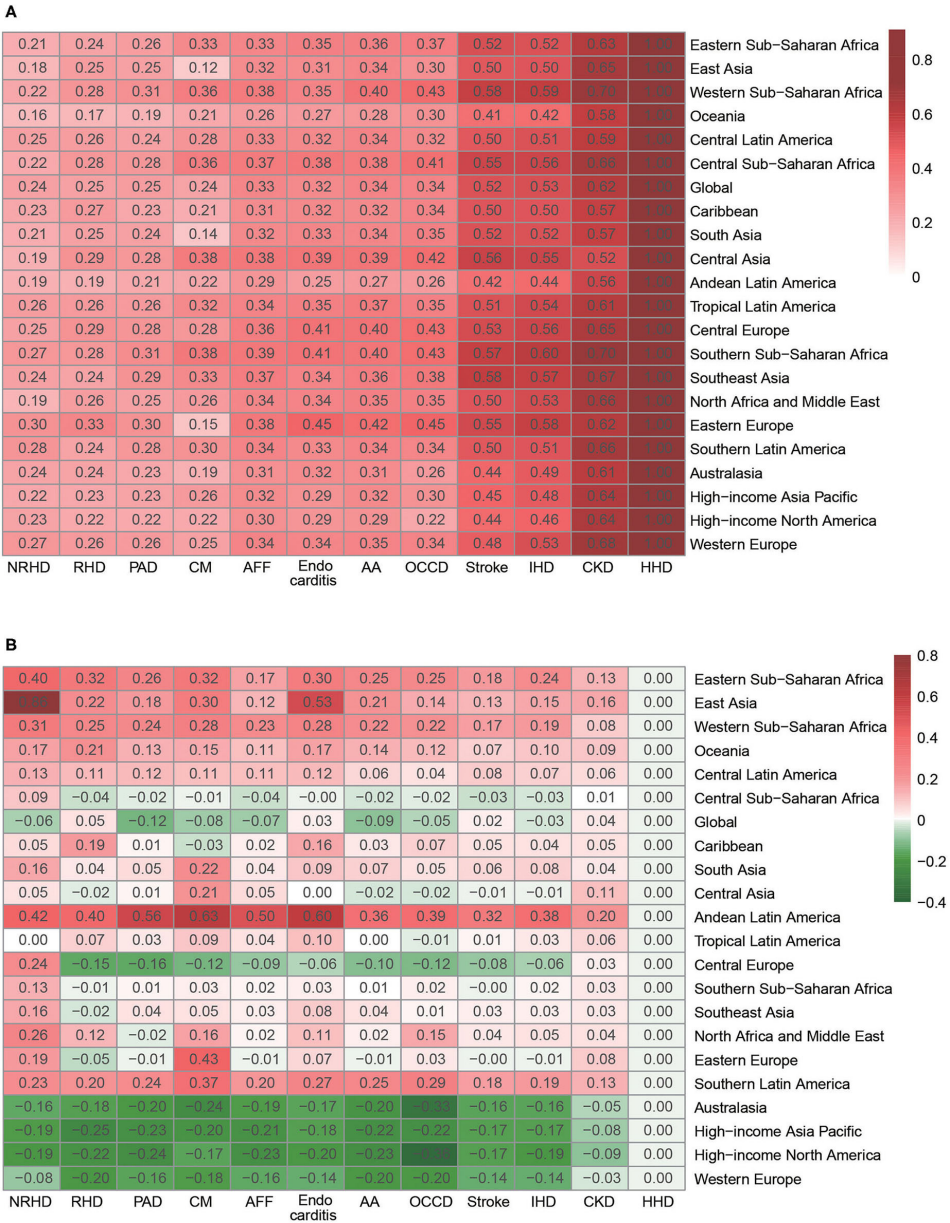
Fraction of disease ASMRs attributable to HSBP by GBD region. **(A)** Fraction of disease ASMRs attributable to HSBP by GBD region in 2019. **(B)** Percentage change in age-standardized population attributable fraction of disease ASMRs attributable to HSBP by GBD region, 1990-2019. ASMRs, age-standardized mortality rates; HSBP, high systolic blood pressure; GBD, global burden of disease, injuries, and risk factors study; AA, aortic aneurysm; AFF, atrial fibrillation and flutter; CM, cardiomyopathy and myocarditis; CKD, chronic kidney disease; HHD, hypertensive heart disease; IHD, ischemic heart disease; NRHD, non-rheumatic valvular heart disease; OCCD, other cardiovascular and circulatory diseases; PAD, peripheral artery disease; RHD, rheumatic heart disease.

Geographically, age-standardized PAFs of most diseases attributed to HSBP in 2019 were relatively low in the four GBD regions with the highest SDI (Australasia, Western Europe, High-income North America, High-income Asia Pacific) while the highest PAFs for most diseases existed commonly in Southern Sub-Saharan Africa and Eastern Europe (Figure 5). In some, Southern Sub-Saharan Africa (low SDI region) had the highest PAF of ASMRs due to HSBP for IHD, CKD, atrial fibrillation and flutter, cardiomyopathy and myocarditis, and PAD, while Eastern Europe (middle SDI region) had the highest PAF of ASMRs imposed by HSBP for AA, other cardiovascular and circulatory diseases, endocarditis, RHD, and non-rheumatic valvular heart disease across 21 GBD regions. The highest PAF of ASMRs due to HSBP for stroke was found in Southeast Asia. In sex subgroups, there were some differences between males and females with different diseases and regions (Supplementary Figure 3). Notably, Southern Sub-Saharan Africa had the highest attributable proportions of ASMRs due to HSBP for CKD, IHD, PAD, and cardiomyopathy and myocarditis in males, and CKD, IHD, cardiomyopathy and myocarditis, atrial fibrillation and flutter, and endocarditis in females. Eastern Europe had the highest age-standardized PAFs of ASMRs due to HSBP for AA, atrial fibrillation and flutter, endocarditis, RHD, other cardiovascular and circulatory diseases, and non-rheumatic valvular heart disease in males and RHD, other cardiovascular and circulatory diseases in females.

Within GBD regions, ASMRs of CVDs and CKD attributable to HSBP varied considerably (Supplementary Figure 4). From 1990 to 2019, the ASMRs for IHD and stroke both in females and in males were still high in 4 GBD regions with middle SDI (Eastern Europe, Central Europe, Central Asia, North Africa and Middle East), especially in Eastern Europe and Central Asia. Moreover, Eastern Europe has the highest ASMR for PAD in both males and females. Africa had high ASMRs for HHD due to HSBP in males and females, especially in Central Sub-Saharan Africa. Alarmingly, uptrends of ASMRs for CKD and endocarditis were observed in most GBD regions, suggesting two urgent problems that must be solved regionally. In some, apparent uptrends of CKD were observed in five GBD regions with low-middle SDI, including Caribbean, Southern Latin America, Central Latin America, Tropical Latin America, and Andean Latin America. Further, four GBD regions with the lowest SDI (Eastern Sub-Saharan Africa, Southern Sub-Saharan Africa, Western Sub-Saharan Africa, Central Sub-Saharan Africa) had relatively high ASMRs of CKD related to HSBP, while 4 GBD regions with the highest SDI had relatively low ASMRs from 1990 to 2019.

Age heterogeneities in PAFs of 12 causes attributed to HSBP were also observed both in males and females (Supplementary Figure 5). The highest PAFs of age-standardized ASMRs due to HSBP for most CVDs were mainly in the age groups of 45-79 years in females and males. In particular, the attributable fraction of ASMRs related to HSBP in endocarditis, RHD, and cardiomyopathy and myocarditis in males were highest in the group aged 25-29 years compared to other age groups. The attributable proportions of CKD ASMRs due to HSBP increased progressively with increasing age in males and females.

In females and males, the ASMRs of 12 causes due to HSBP were less pronounced at a younger age but increased with age (Supplementary Figure 6). The trends of ASMRs of 12 causes imposed by HSBP in each age group were similar in males and females. Between 1990 and 2019, ASMRs of 12 causes attributable to HSBP were consistently highest in the >95 year age group, except for AA in the male group. In contrast, the lowest ASMRs of 12 causes related to HSBP from 1990 to 2019 were observed for the age <20 years and 20-24 years groups. Surprisingly, the trends of ASMRs for IHD, stroke, and RHD during the study period declined substantially in each age group, except for <20 and 20-24 age groups, which had no data or data with 0. ASMRs of CKD, HHD, endocarditis, and non-rheumatic valvular heart disease due to HSBP were distinctly increased in the 90-94 and 95 plus years age groups from 1990 to 2019.

## Discussion

This analysis revealed a substantial decline in the HSBP-associated mortality burden after years of blood pressure control efforts. However, HSBP-induced disability was not successfully controlled at the global level, which is worth emphasizing and indicates the need to implement concrete measures. HSBP related to disease burden and the reduction in the disease burden associated with SBP control vary among different diseases and SDI regions. HHD, CKD, IHD, and stroke are the most common diseases related to HSBP-related death and have not been well-controlled globally, except in high SDI regions. The HSBP-associated death burden from atrial fibrillation and flutter, PAD, and endocarditis is increasing in low to middle SDI regions. In addition, the contributions of HSBP to diseases differed according to age and sex. Given the large variations in the HSBP-related burden of disease by region, sex, and age, strategies to reduce the HSBP-associated burden should be developed and implemented. To the best of our knowledge, our study is the first to reveal all cause-specific burdens attributable to HSBP using GBD 2019 data.

HSBP burden discrepancies at SDI, regional, and national level can be explained by the economic level, medical level, prevention and control policy, education level and awareness, degree of population aging, lifestyle and diet, gene susceptibility, environment, etc. Awareness, treatment, and control rates of HSBP in high-SDI countries are quite higher in comparison with low- and middle-SDI countries (20). Successful strategies used to address HSBP-related burdens in high-SDI regions, such as the United States, could provide a policy reference for other regions. The United States has achieved a great improvement in blood pressure control, due to better community-based interventions, increased awareness of blood pressure control, increased blood pressure treatment rates, and extensive implementation of the updated guidelines for hypertension (12, 14, 21). Despite these dramatic improvements, high SDI regions should note that most disease burdens attributed to HSBP remained stable or even increased in recent years. Recent studies from high-income countries have shown that control rates of hypertension have plateaued and even declined in the past few years (22, 23), which may in part explain our findings. Policymakers in high-income regions should further analyze the causes of stagnation and aim to optimize existing policies. Notably, although the high SDI-associated death burden achieved a marked reduction from 1990 to 2019, the disease burden from high SDI-induced disability is still predominant and has not been adequately controlled. This finding implies that public health policymakers should consider improving the quality of life of patients with HSBP.

Low/low-middle/middle SDI regions, on the other hand, exhibited unchanged or increasing disease burdens associated with HSBP due to low awareness and limited resources for prevention, screening, and intervention (24, 25). More importantly, the PAFs of disease mortality due to HSBP increased from 1990 to 2019. Without effective interventions, the increasing burden will continue to exacerbate the CVD and CKD epidemics, disabilities, and deaths. For example, population aging, urbanization, and increasingly Westernized lifestyles have led to an increased risk of HSBP in China. However, basic medical insurance was not yet complete, and the awareness, treatment, and control rates of hypertension are low, which has greatly increased the burden of HSBP (26). Although evidence-based interventions are well-known for management of hypertension and related diseases, implementation of interventions in these regions faces pernicious challenges at a population level (25, 27, 28). Considering the limited resources, successful control of HSBP in these regions should entail a comprehensive strategy of raising awareness at population levels and lifestyle modification at individual levels (5). Emerging evidence supports cost-effective strategies to control hypertension at the community, healthcare, and population levels in lower SDI regions (29, 30). At the community level, several novel and innovative strategies have been implemented and received great achievements in low-income countries, such as South Africa, Bangladesh, and Pakistan, including mobilizing communities to participate in health care services, educating community members to raise awareness of hypertension, screening patients in their homes or community settings, and delivering home or community-based lifestyle, social, or environmental interventions (30–33). At the healthcare level, consistent and reliable base health care with affordable service is conducive to improving the awareness, prevention, treatment, control, and compliance of hypertension. At the population level, salt reduction and tobacco control were associated with better hypertension control and sufficient cardiovascular disease prevention (30, 34–36). Therefore, it can be considered that in low-income countries, multi-component comprehensive strategy can be taken from the community, medical care, population, to have a wide-ranging impact on the prevention, treatment, and control of HSBP.

Notably, global ASMRs of CKD and endocarditis due to HSBP presented uptrends, calling for enhanced prevention and control. HSBP is one of major risk factors for CKD that can be present in the earliest stages of CKD and is well-documented to contribute to cardiovascular morbidity and mortality (37). Since patients with early stages of CKD are usually asymptomatic and later stages of CKD may lead to severe sequelae and a poor prognosis, earlier detection and timely treatment of kidney impairment should be emphasized in patients with HSBP to slow CKD deterioration. While the optimal SBP for minimizing the risk of CKD progression and mortality remains unclear (38), clinical trials based on large population are necessary. Another disease that has not improved substantially in all SDI regions is endocarditis, which has received far less global attention. With aging society, the wide use of implanted electronic devices for CVDs, and opioid-associated drug injections, the global incidence of endocarditis has been sharply increased in recent years (39, 40). Increasing burden of HSBP was related to CVDs, diabetes, and cancer, all of which may contribute to the rising burden of endocarditis (12, 41–43). Unfortunately, significant gaps exist in the knowledge of a causal relationship between HSBP and endocarditis; therefore, it is urgent to carry out in-depth studies and take measures to reverse this unfavorable trend.

Males had higher ASMRs of all causes and most specific diseases due to HSBP than females. Sex disparities in disease burden mirror inherent mechanical discrepancies that regulate blood pressure. For example, increased longevity in females and the cardioprotective effects of estrogen may limit organ damage caused by HSBP (44). Males are exposed to more social and environmental risks, such as smoking, drinking, poor eating habits, etc., which may also lead to gender differences in the burden of HSBP. Importantly, males have lower levels of hypertension awareness and lower rates of antihypertensive treatment compared with females. Correspondingly, strict control of blood pressure and related risk factors by improving males' lifestyles or improving health awareness is essential to reduce the overall mortality of HSBP patients. Notably, ASMRs of IHD, AA, and non-rheumatic valvular heart disease in males have declined more quickly than those in females in high SDI regions since 1990. Understanding the precise reason for this reduction may benefit the global population by providing information related to controlling SBP and reducing related disease burdens.

There was substantial heterogeneity in disease burdens attributed to HSBP by age. The 45-79 year group was the main group with high CVDs ASMRs attributed to HSBP in both females and males, suggesting that middle-aged people may be more affected by HSBP (45). However, people aged 80 plus years had the highest ASMRs of CVDs related to HSBP. Reasons for the age heterogeneity are multifactorial. On the one hand, arterial aging, arterial stiffness, and vasoconstriction dysfunction associated with aging, lead to a sharp increase in the prevalence and poor prognosis of HSBP and cardiovascular disease with age (46, 47). On the other hand, the age disparity in the awareness, treatment and control of hypertension is another important reason. Excitingly, the recent National Health and Nutrition Examination Survey revealed a greater improvement in awareness and treatment of hypertension in younger adults (48). There is no doubt that high awareness, timely detection, and effective treatment from a younger age can greatly improve the prevalence and prognosis of HSBP. Meanwhile, people aged above 45 years old are the major target population for screening HSBP-associated CVD. Notably, HSBP is more likely associated with the disease burden of endocarditis, RHD, non-rheumatic valvular heart disease, and cardiomyopathy and myocarditis related disease burden in the population aged 25-29 years. In older patients aged above 50 years, HSBP is the predominant risk factor for CKD mortality. These findings highlight that HSBP-related disease burden has age-specific characteristics, which indicates the need for adopting age-specific screening for organ damage in individuals with HSBP.

Our research has several limitations. First, the major limitation of the GBD analysis, as described in other GBD studies, is the availability of primary data and representativeness of partial samples for the entire territory/country, which influence the integrity and accuracy of data. Second, GBD 2019 adjusted its data sources, collation, and analytical strategies to decrease missing data and improve its data quality and comparability, which may bias the results. Third, different access to SBP testing methods and CVD diagnostic technologies, as well as discrepant diagnostic standards, may influence estimates of some conditions. Fourth, our study was conducted at the global and regional levels without further analyzing discrepancies in countries and domestic areas. Finally, we could not access the data on the burden of heart failure, coronary heart diseases, and heart arrest attributed to HSBP from GBD 2019. Thus, future research is warranted to verify the results of this study.

## Conclusion

Although there was a substantial decline in the HSBP-associated death burden from 1990 to 2019, HSBP-induced disability was not successfully controlled at the global level, which is worth emphasizing and requires the implementation of concrete measures. HSBP was the leading risk factor attributed to CVDs and CKD mortality worldwide and the cause-specific burden related to HSBP varied by region, age, and sex. The disease burden related to HSBP was higher in low and low-middle SDI regions than in higher SDI regions in 2019. The downtrends of HSBP-related burden in high SDI regions have flattened in recent years. Given the large variations in the HSBP-related burden of diseases by region, sex, and age, strategies to reduce the HSBP-associated burden should be developed and implemented differently according to differences associated with these characteristics.

## Data Availability Statement

The original contributions presented in the study are included in the article/Supplementary Material, further inquiries can be directed to the corresponding author/s.

## Author Contributions

M-MC and XZ designed study, analyzed data, and wrote the first draft. Y-ML, ZC, and HaL collected data and contributed to data analysis. FL, J-JQ, YJ, PZ, JC, Z-GS, and X-JZ revised the manuscript. HuL, ZL, and HoL contributed equally, designed the project, edited manuscript, and supervised the study. All authors have approved the final version of this paper.

## Funding

This work was supported by grants from the National Science Foundation of China (81630011, 81970364, 81970070, 81970011, 81770053, 81870171, 82000299), the Hubei Science and Technology Support Project (2019BFC582, 2018BEC473), and Medical flight plan of Wuhan University (TFJH2018006).

## Conflict of Interest

The authors declare that the research was conducted in the absence of any commercial or financial relationships that could be construed as a potential conflict of interest.

## Publisher's Note

All claims expressed in this article are solely those of the authors and do not necessarily represent those of their affiliated organizations, or those of the publisher, the editors and the reviewers. Any product that may be evaluated in this article, or claim that may be made by its manufacturer, is not guaranteed or endorsed by the publisher.
