# Supplementary material for: Heavy Disease Burden of High Systolic Blood Pressure During 1990-2019: Highlighting Regional, Sex, and Age Specific Strategies in Blood Pressure Control
Source: Front Cardiovasc Med. 2021 Dec 16;8:754778. doi: 10.3389/fcvm.2021.754778 (PMC8716441; doi:10.3389/fcvm.2021.754778)
Supplement: Supplementary file 1 [file Data_Sheet_1.pdf]

Supplemental Material

MM Chen, X Zhang *et al.* The heavy disease burden of high systolic blood pressure during 1990-2019: Highlighting regional, sex, and age specific strategies in blood pressure control

Table S1. ASDRs and age-standardized rate of YLLs attributable to HSBP by sex, SDI regions in 1990, 2019 and EAPC from 1990 to 2019

Table S2. ASMRs and ASYRs attributable to HSBP by GBD regions in 1990, 2019 and EAPC from 1990 to 2019

Table S3. ASDRs and age-standardized rate of YLLs attributable to HSBP by GBD regions in 1990, 2019 and EAPC from 1990 to 2019

Table S4. ASMRs and ASYRs attributable to HSBP by 204 countries in 1990, 2019 and EAPC from 1990 to 2019.

Table S5. 21 GBD regions and their country compositions

Figure S1. Age-specific rate of deaths and YLDs attributable to HSBP in 1990-2019

Figure S2. ASMRs and ASYRs attributable to HSBP across 204 countries and territories by socio-demographic index for both sexes combined in 2019

Figure S3. Fraction of disease ASMRs attributable to HSBP by GBD region for female and male in 2019

Figure S4. ASMRs of 12 causes attributable to HSBP across GBD regions for female and male, 1990-2019.

Figure S5. Fraction of disease ASMRs attributable to HSBP by age group in 2019.

Figure S6. ASMRs of 12 causes attributable to HSBP for female and male by age, 1990-2019.

Table S1. ASDRs and age-standardized rate of YLLs attributable to HSBP by sex, SDI regions in 1990, 2019 and EAPC from 1990 to 2019.

Characteristics	1990	2019	1990-2019	1990	2019	1990-2019
	**ASDRs per 100000 (95 % UI)**	**ASDRs per 100000 (95 % UI)**	**EAPC (95 % CI)**	**Age-standardized rate of YLLs per 100000 (95 % UI)**	**Age-standardized rate of YLLs per 100000 (95 % UI)**	**EAPC (95 % CI)**
**Global**						
Overall	3953.92(3557.53,4359.13)	2885.57(2580.75,3201.05)	-1.17(-1.22, -1.13)	3697.88(3323.64,4072.43)	2627.03(2343.52,2900.80)	-1.28(-1.33,-1.22)
Male	4538.11(4060.32,5008.08)	3448.86(3060.06,3837.69)	-0.99(-1.03, -0.94)	4292.05(3827.50,4726.88)	3194.79(2814.31,3567.79)	-1.07(-1.11,-1.02)
Female	3403.35(3025.28,3766.52)	2354.72(2075.57,2634.68)	-1.41(-1.47, -1.35)	3139.95(2795.92,3481.57)	2093.10(1827.11,2357.98)	-1.56(-1.62,-1.49)
**SDI**						
High SDI	3030.94(2717.97,3318.13)	1385.57(1222.65,1545.55)	-3.03(-3.25, -2.81)	2768.38(2480.07,3022.65)	1176.18(1036.86,1305.41)	-3.32(-3.55,-3.09)
High-middle SDI	4484.56(4045.68,4918.02)	2845.57(2528.64,3172.17)	-1.86(-2.09, -1.64)	4204.78(3781.30,4593.91)	2570.46(2274.98,2867.11)	-2.02(-2.26,-1.77)
Middle SDI	4077.97(3616.32,4559.38)	3383.27(3009.74,3767.07)	-0.51(-0.57, -0.44)	3820.00(3373.75,4264.48)	3083.02(2725.06,3452.02)	-0.60(-0.67,-0.53)
Low-middle SDI	3984.95(3515.85,4487.43)	3614.21(3195.63,4062.85)	-0.32(-0.37, -0.27)	3787.57(3330.08,4264.11)	3388.16(2981.85,3816.91)	-0.37(-0.43,-0.32)
Low SDI	4147.00(3621.05,4740.15)	3682.42(3212.75,4153.97)	-0.40(-0.47, -0.34)	3958.42(3443.22,4545.55)	3473.86(3029.88,3918.62)	-0.45(-0.52,-0.38)

ASDRs, age-standardized rate of disability-adjusted life years; YLL, years of life lost; EAPC, estimated annual percentage change; UI, uncertainty interval; CI, confidence interval; SDI, sociodemographic index.

Table S2. ASMRs and ASYRs attributable to HSBP by GBD regions in 1990, 2019 and EAPC from 1990 to 2019.

Characteristics	1990 ASMRs per 100000 (95 % UI)	2019 ASMRs per 100000 (95 % UI)	1990-2019 EAPC (95 % CI)	1990 ASYRs per 100000 (95 % UI)	2019 ASYRs per 100000 (95 % UI)	1990-2019 EAPC (95 % CI)
**GBD region**						
High-income Asia Pacific	130.47(112.58,145.62)	41.90(33.84,48.79)	-4.19(-4.39,-3.99)	271.09(195.40,346.74)	193.92(138.25,250.38)	-1.39(-1.51,-1.27)
High-income North America	148.69(126.09,168.89)	78.66(65.78,90.81)	-2.64(-2.88,-2.40)	275.63(198.66,355.91)	233.80(169.10,303.20)	-0.56(-0.66,-0.46)
Western Europe	169.41(146.62,189.59)	70.67(59.21,80.20)	-3.37(-3.57,-3.17)	230.93(165.84,299.01)	165.95(118.84,217.40)	-1.27(-1.35,-1.19)
Australasia	158.42(134.59,179.68)	56.22(45.36,66.93)	-4.06(-4.33,-3.80)	225.85(161.35,293.81)	162.35(115.59,215.47)	-1.24(-1.32,-1.15)
Eastern Europe	311.30(268.29,352.12)	256.06(213.75,295.04)	-1.21(-1.65,-0.77)	327.31(235.90,417.15)	298.27(214.50,383.91)	-0.34(-0.35,-0.33)
Central Europe	317.12(276.44,351.87)	177.90(147.08,209.58)	-2.30(-2.44,-2.16)	368.06(265.21,472.79)	290.53(207.39,372.82)	-0.77(-0.82,-0.73)
Southern Latin America	147.28(123.36,171.63)	101.13(87.67,113.57)	-1.17(-1.25,-1.10)	158.62(113.38,209.53)	169.57(122.95,221.72)	0.43(0.32,0.55)
East Asia	190.13(159.49,224.32)	151.15(125.08,179.46)	-0.52(-0.64,-0.40)	256.82(182.53,342.73)	322.67(230.11,419.79)	0.97(0.92,1.01)
Central Asia	297.71(259.62,336.54)	335.07(282.32,382.09)	0.13(-0.27,0.53)	303.25(218.48,387.18)	292.18(210.10,375.12)	-0.14(-0.17,-0.10)
North Africa and Middle East	286.51(246.73,323.37)	219.41(185.61,252.54)	-0.97(-1.01,-0.94)	275.88(198.31,360.24)	310.90(225.31,401.33)	0.44(0.36,0.51)
Southeast Asia	206.98(181.24,232.52)	191.47(165.93,214.85)	-0.11(-0.23,0.00)	337.93(244.23,429.80)	364.61(263.39,466.83)	0.33(0.29,0.37)
Southern Sub-Saharan Africa	163.27(143.90,183.	173.87(154.25,193.	0.25(-0.20,0.71)	264.95(191.79,342	274.18(199.86,350	0.04(-0.02,0.10)

	22)	42)		.70)	.55)	
Tropical Latin America	195.69(172.95,216.97)	105.21(91.35,116.71)	-2.16(-2.22,-2.10)	220.40(158.13,285.70)	199.83(145.06,259.66)	-0.41(-0.44,-0.37)
Andean Latin America	85.80(70.22,101.84)	76.56(60.96,93.41)	-0.02(-0.27,0.23)	93.69(66.47,125.18)	136.83(98.53,179.02)	1.50(1.37,1.63)
Caribbean	173.40(149.70,196.26)	139.47(116.65,164.42)	-0.66(-0.83,-0.50)	180.03(130.51,235.03)	207.63(150.35,272.39)	0.55(0.53,0.57)
Central Latin America	130.36(113.44,147.02)	112.97(94.00,133.25)	-0.56(-0.64,-0.47)	193.19(139.24,254.64)	226.35(162.34,294.21)	0.58(0.52,0.63)
South Asia	175.50(149.69,202.76)	151.43(128.27,174.84)	-0.58(-0.70,-0.45)	169.86(122.87,221.13)	185.58(134.01,240.64)	0.47(0.39,0.54)
Central Sub-Saharan Africa	250.74(213.22,291.64)	212.90(173.49,259.07)	-0.63(-0.75,-0.52)	219.17(160.97,282.92)	203.92(148.54,262.77)	-0.36(-0.40,-0.31)
Oceania	178.71(146.03,218.68)	190.92(152.37,240.19)	0.25(0.15,0.34)	260.14(184.88,339.07)	311.43(222.47,406.36)	0.63(0.50,0.77)
Western Sub-Saharan Africa	176.18(145.43,212.39)	174.92(146.23,202.29)	0.12(0.00,0.24)	199.79(145.49,261.12)	254.28(186.33,325.44)	1.08(0.99,1.18)
Eastern Sub-Saharan Africa	180.60(152.72,208.62)	171.38(148.97,193.63)	-0.20(-0.22,-0.17)	199.18(145.40,260.94)	235.72(172.17,307.27)	0.63(0.62,0.65)

ASMRs, age-standardized mortality rate; ASYRs, age-standardized rates of years lived with disability; EAPC, estimated annual percentage change; UI, uncertainty interval; CI, confidence interval.

Table S3. ASDRs and age-standardized rate of YLLs attributable to HSBP by GBD regions in 1990, 2019 and EAPC from 1990 to 2019.

Characteristics	1990 ASDRs per 100000 (95 % UI)	2019 ASDRs per 100000 (95 % UI)	1990-2019 EAPC (95 % CI)	1990 Age-standardized YLLs per 100000 (95 % UI)	2019 Age-standardized YLLs per 100000 (95 % UI)	1990-2019 EAPC (95 % CI)
**GBD region**						
High-income Asia Pacific	2435.39(2178.24,2661.06)	877.85(763.25,997.03)	-3.79(-3.96,-3.62)	2164.31(1936.59,2352.42)	683.93(591.16,778.46)	-4.26(-4.44,-4.09)
High-income North America	2886.77(2564.19,3210.37)	1612.08(1416.47,1801.45)	-2.37(-2.63,-2.12)	2611.14(2317.33,2901.43)	1378.28(1209.99,1544.80)	-2.62(-2.89,-2.34)
Western Europe	3057.88(2756.59,3322.48)	1209.14(1059.49,1339.75)	-3.56(-3.77,-3.35)	2826.96(2543.83,3073.75)	1043.19(907.82,1155.41)	-3.83(-4.05,-3.61)
Australasia	2897.47(2574.26,3212.57)	1005.82(859.88,1149.81)	-4.07(-4.36,-3.78)	2671.62(2368.10,2952.91)	843.46(716.71,960.95)	-4.45(-4.76,-4.14)
Eastern Europe	5995.64(5386.27,6535.37)	5068.18(4334.26,5751.43)	-1.19(-1.68,-0.68)	5668.34(5074.37,6211.57)	4769.91(4068.54,5416.35)	-1.23(-1.75,-0.70)
Central Europe	6174.40(5620.42,6676.45)	3239.08(2770.97,3726.76)	-2.57(-2.71,-2.44)	5806.33(5287.91,6262.54)	2948.55(2495.62,3411.82)	-2.71(-2.86,-2.56)
Southern Latin America	2841.58(2431.12,3243.61)	1900.09(1690.29,2110.60)	-1.28(-1.35,-1.21)	2682.96(2298.77,3062.49)	1730.51(1542.98,1917.66)	-1.41(-1.48,-1.34)
East Asia	3677.49(3087.12,4279.16)	2824.92(2377.72,3288.85)	-0.70(-0.80,-0.60)	3420.67(2858.88,4017.11)	2502.26(2055.28,2942.01)	-0.86(-0.97,-0.75)
Central Asia	6215.94(5619.84,6832.55)	6429.35(5614.52,7222.91)	-0.22(-0.60,0.17)	5912.69(5348.30,6489.10)	6137.17(5331.83,6919.35)	-0.22(-0.62,0.18)
North Africa and Middle East	5887.94(5221.21,6582.29)	4401.89(3785.93,5042.84)	-1.08(-1.12,-1.05)	5612.06(4963.12,6311.96)	4090.99(3504.40,4730.84)	-1.18(-1.22,-1.14)
Southeast Asia	4545.47(4053.62,5043.51)	4140.21(3660.38,4624.23)	-0.16(-0.26,-0.06)	4207.53(3761.01,4685.84)	3775.60(3312.18,4240.42)	-0.20(-0.31,-0.09)

Southern Sub-Saharan Africa	3598.91(3218.01,3981.67)	3485.51(3148.97,3850.10)	-0.08(-0.53,0.37)	3333.96(2966.42,3704.93)	3211.32(2886.91,3561.45)	-0.09(-0.57,0.39)
Tropical Latin America	4246.22(3832.93,4636.15)	2289.89(2058.57,2502.64)	-2.19(-2.24,-2.14)	4025.82(3631.91,4399.07)	2090.07(1874.78,2281.43)	-2.32(-2.37,-2.27)
Andean Latin America	1728.39(1435.20,2046.52)	1465.56(1182.68,1784.36)	-0.29(-0.54,-0.03)	1634.70(1356.29,1934.89)	1328.72(1057.33,1631.58)	-0.42(-0.69,-0.16)
Caribbean	3666.64(3271.80,4096.40)	3108.25(2602.39,3643.56)	-0.49(-0.65,-0.32)	3486.61(3088.32,3899.80)	2900.62(2416.40,3437.83)	-0.55(-0.73,-0.38)
Central Latin America	2629.12(2338.29,2904.85)	2348.24(2002.96,2736.06)	-0.45(-0.53,-0.37)	2435.94(2175.91,2686.43)	2121.90(1780.71,2494.71)	-0.54(-0.63,-0.46)
South Asia	3827.95(3331.09,4365.49)	3358.83(2897.10,3852.19)	-0.45(-0.55,-0.35)	3658.09(3174.70,4176.06)	3173.25(2716.09,3659.01)	-0.50(-0.61,-0.39)
Central Sub-Saharan Africa	5395.27(4570.65,6334.03)	4359.22(3552.74,5326.98)	-0.82(-0.93,-0.71)	5176.10(4353.34,6115.43)	4155.30(3343.41,5124.66)	-0.84(-0.95,-0.72)
Oceania	4262.48(3497.63,5172.45)	4699.37(3717.62,5885.74)	0.40(0.26,0.55)	4002.34(3249.56,4912.90)	4387.94(3439.96,5556.67)	0.39(0.24,0.53)
Western Sub-Saharan Africa	3650.92(3040.04,4382.73)	3581.85(3026.98,4125.16)	0.08(-0.04,0.19)	3451.12(2855.95,4203.97)	3327.57(2770.58,3867.67)	0.01(-0.11,0.13)
Eastern Sub-Saharan Africa	3920.39(3345.83,4522.83)	3560.74(3106.51,4018.66)	-0.37(-0.41,-0.34)	3721.21(3156.18,4301.50)	3325.02(2882.35,3769.69)	-0.43(-0.47,-0.40)

ASDRs, age-standardized disability-adjusted life years; YLL, years of life lost; EAPC, estimated annual percentage change; UI, uncertainty interval; CI, confidence interval

Table S4. ASMRs and ASYRs attributable to HSBP by 204 countries in 1990, 2019 and EAPC from 1990 to 2019.

SDI region	Countries	1990 ASMRs per 100000(95 % UI)	2019 ASMRs per 100000(95 % UI)	1990-2019 EAPC (95 % CI)	1990 ASYRs per 100000(95 % UI)	2019 ASYRs per 100000(95 % UI)	1990-2019 EAPC (95 % CI)
Low SDI	Somalia	219.66(167.52,276.68)	220.25(169.70,286.75)	0.21(0.12,0.29)	199.43(140.87,265.98)	219.24(158.45,291.12)	0.37(0.34,0.41)
Low SDI	Niger	185.25(145.89,231.80)	172.83(136.88,212.52)	-0.20(-0.26,-0.13)	200.41(143.87,262.02)	217.79(155.24,285.97)	0.32(0.30,0.34)
Low SDI	Chad	163.95(131.52,202.44)	174.16(139.69,214.62)	0.30(0.16,0.43)	186.54(134.37,243.46)	210.19(152.40,273.72)	0.43(0.41,0.44)
Low SDI	Burkina Faso	149.05(119.32,181.92)	175.78(142.11,209.52)	0.81(0.66,0.96)	168.88(120.43,220.80)	184.33(132.56,243.72)	0.27(0.21,0.33)
Low SDI	Mali	177.05(144.39,213.96)	162.25(127.74,199.76)	-0.26(-0.33,-0.19)	179.55(126.92,236.97)	190.63(136.22,250.68)	0.19(0.11,0.26)
Low SDI	Central African Republic	299.15(241.93,364.89)	283.16(214.39,367.31)	-0.15(-0.18,-0.12)	211.90(154.33,273.64)	216.06(156.76,280.95)	0.07(0.05,0.08)
Low SDI	Burundi	223.45(167.83,286.59)	186.43(146.81,233.13)	-0.82(-0.90,-0.74)	225.41(162.31,297.93)	226.66(164.22,297.39)	0.02(0.00,0.04)
Low SDI	Mozambique	203.27(166.46,245.41)	241.61(192.72,301.41)	0.94(0.80,1.09)	253.38(184.92,329.41)	311.58(229.89,404.04)	0.79(0.73,0.86)
Low SDI	Guinea	161.81(130.74,197.29)	182.17(144.54,224.99)	0.78(0.65,0.91)	178.91(129.78,239.82)	224.28(161.97,294.19)	0.96(0.88,1.05)
Low SDI	Afghanistan	405.00(319.87,490.75)	341.80(262.19,421.64)	-0.71(-0.83,-0.58)	245.40(175.66,319.26)	279.24(204.03,362.76)	0.50(0.42,0.58)
Low SDI	Ethiopia	152.73(115.38,194.95)	120.88(101.56,141.15)	-0.95(-1.05,-0.86)	113.08(80.30,150.29)	170.11(121.26,225.48)	1.63(1.49,1.78)
Low SDI	Sierra Leone	229.28(180.99,285.	210.94(161.76,265.	-0.08(-0.20,0.0	291.62(211.95,373.	306.34(224.39,395.	0.17(0.13,0.21

		92)	78)	5)	51)	90)	)
Low SDI	Benin	185.75(153.33,219.72)	178.35(141.62,220.47)	-0.09(-0.15,-0.03)	205.62(147.99,267.19)	239.71(173.99,311.94)	0.54(0.46,0.62)
Low SDI	Guinea-Bissau	246.72(195.76,300.93)	247.94(195.79,306.52)	0.21(0.11,0.31)	234.72(170.52,308.46)	264.49(190.32,343.34)	0.45(0.40,0.49)
Low SDI	South Sudan	170.58(130.79,214.27)	154.15(117.89,196.80)	-0.30(-0.39,-0.20)	217.48(161.00,290.63)	253.61(185.10,333.60)	0.59(0.55,0.63)
Low SDI	Liberia	192.30(158.50,228.42)	172.35(131.11,219.30)	-0.23(-0.35,-0.10)	220.00(159.24,285.16)	240.19(174.74,313.52)	0.47(0.39,0.54)
Low SDI	Democratic Republic of the Congo	244.24(203.79,287.85)	208.01(165.48,258.35)	-0.59(-0.72,-0.45)	211.65(154.97,274.40)	186.64(135.42,244.54)	-0.59(-0.65,-0.52)
Low SDI	Malawi	190.64(156.64,226.40)	182.29(149.27,215.67)	-0.29(-0.47,-0.11)	250.28(181.11,328.59)	278.34(201.80,362.93)	0.35(0.26,0.44)
Low SDI	Senegal	199.79(165.28,233.09)	182.98(146.24,224.60)	-0.16(-0.29,-0.03)	266.76(193.64,346.55)	261.89(190.75,339.16)	-0.06(-0.16,0.04)
Low SDI	Papua New Guinea	132.44(97.93,174.85)	154.07(112.06,206.99)	0.68(0.49,0.87)	211.52(147.14,281.38)	260.24(184.21,342.05)	0.74(0.55,0.93)
Low SDI	Eritrea	193.10(134.78,275.88)	195.26(146.00,259.02)	0.00(-0.05,0.05)	184.22(131.44,245.02)	194.21(139.75,261.87)	0.15(0.12,0.17)
Low SDI	Madagascar	231.31(193.46,270.92)	255.82(198.25,322.00)	0.24(0.09,0.39)	250.47(179.23,324.43)	293.67(210.92,382.08)	0.58(0.51,0.66)
Low SDI	Gambia	184.58(145.23,228.12)	217.26(171.27,267.04)	0.60(0.46,0.74)	240.92(174.15,314.63)	277.82(200.89,362.44)	0.55(0.46,0.64)
Low SDI	Uganda	177.84(143.09,212.35)	163.33(131.69,193.61)	-0.64(-0.94,-0.34)	240.05(173.76,312.15)	228.70(165.74,299.96)	-0.27(-0.45,-0.09)
Low SDI	Solomon Islands	306.24(229.28,396.32)	332.78(256.40,418.60)	0.20(0.12,0.28)	271.00(186.30,362.32)	298.67(210.98,393.87)	0.17(0.08,0.26)
Low SDI	Cote d'Ivoire	211.07(174.00,251.	190.18(149.99,232.	-0.38(-0.56,-0.	256.08(185.31,329.	275.31(200.84,357.	0.18(0.10,0.26

		22)	30)	20)	89)	01)	)
Low SDI	Yemen	320.82(250.15,401.62)	280.08(216.95,353.98)	-0.58(-0.64,-0.52)	237.16(167.35,311.82)	276.79(199.16,361.40)	0.64(0.60,0.68)
Low SDI	Togo	205.38(169.26,245.67)	199.78(159.33,248.22)	0.00(-0.13,0.14)	246.99(177.43,320.98)	257.60(186.21,336.37)	0.14(0.05,0.23)
Low SDI	Nepal	126.43(98.36,165.48)	120.13(91.86,148.61)	0.02(-0.09,0.13)	129.84(92.70,174.06)	138.97(98.01,183.46)	0.24(0.04,0.44)
Low SDI	United Republic of Tanzania	175.27(148.84,204.35)	189.87(158.09,222.07)	0.45(0.32,0.57)	199.08(145.26,263.12)	284.68(206.52,370.76)	1.47(1.34,1.60)
Low SDI	Rwanda	239.90(184.76,304.94)	154.89(126.42,190.45)	-2.15(-2.43,-1.88)	229.60(163.00,300.11)	197.16(142.30,261.75)	-0.63(-0.71,-0.54)
Low SDI	Haiti	324.09(263.59,411.12)	281.86(208.00,374.71)	-0.26(-0.39,-0.14)	204.23(148.45,265.80)	248.24(180.26,322.09)	0.89(0.84,0.95)
Low SDI	Pakistan	187.30(150.45,225.01)	225.69(186.22,274.91)	0.51(0.32,0.70)	211.84(152.67,278.32)	251.72(182.04,325.31)	0.65(0.53,0.77)
Low-middle SDI	Bhutan	142.83(106.85,181.89)	141.11(110.35,174.15)	0.04(-0.03,0.11)	154.05(111.53,203.58)	178.24(126.94,231.66)	0.62(0.54,0.70)
Low-middle SDI	Comoros	201.33(141.10,253.95)	171.78(140.71,210.80)	-0.70(-0.82,-0.59)	274.66(195.63,361.13)	265.46(192.16,348.20)	-0.19(-0.21,-0.16)
Low-middle SDI	Djibouti	178.48(138.20,223.01)	186.20(147.61,232.35)	0.14(0.08,0.19)	229.54(163.28,303.21)	273.39(198.71,357.50)	0.70(0.65,0.74)
Low-middle SDI	Cambodia	191.30(156.61,232.72)	149.39(117.79,179.44)	-1.08(-1.18,-0.97)	249.98(176.94,331.94)	219.46(154.71,292.13)	-0.68(-0.77,-0.59)
Low-middle SDI	Angola	242.87(192.02,300.75)	207.92(170.25,254.07)	-0.73(-0.85,-0.61)	234.67(171.42,304.55)	238.79(173.81,309.64)	0.03(0.00,0.05)
Low-middle SDI	Zimbabwe	180.05(151.33,213.64)	220.99(173.79,280.48)	1.23(0.92,1.54)	198.52(144.54,260.05)	245.79(177.51,314.24)	0.71(0.58,0.84)
Low-middle SDI	Bangladesh	161.70(131.15,192.62)	155.43(117.16,193.11)	0.15(-0.08,0.38)	149.68(105.67,197.43)	196.87(142.33,256.80)	1.06(0.83,1.28)

Low-middle SDI	Vanuatu	341.35(265.93,430.33)	382.05(300.67,481.38)	0.19(0.07,0.30)	485.43(347.57,620.49)	512.30(368.66,659.41)	0.08(0.01,0.15)
Low-middle SDI	Cameroon	154.71(125.68,185.77)	189.97(151.28,238.48)	0.87(0.40,1.35)	159.69(115.92,211.67)	255.45(184.92,334.21)	1.82(1.37,2.27)
Low-middle SDI	Lao People's Democratic Republic	267.17(208.78,330.65)	222.52(177.16,270.67)	-0.81(-0.92,-0.70)	349.89(246.32,455.53)	317.55(230.01,416.75)	-0.46(-0.49,-0.42)
Low-middle SDI	Honduras	134.68(109.42,176.27)	189.18(154.82,229.48)	1.33(1.11,1.55)	163.33(115.99,216.02)	195.69(141.04,256.42)	0.57(0.55,0.60)
Low-middle SDI	Mauritania	214.29(178.62,253.83)	149.50(120.07,181.68)	-1.16(-1.26,-1.07)	258.82(186.40,339.85)	254.70(184.97,331.70)	-0.10(-0.16,-0.05)
Low-middle SDI	Sao Tome and Principe	185.19(152.88,217.17)	223.72(176.28,261.96)	0.66(0.54,0.79)	301.78(217.31,396.32)	359.21(263.52,463.25)	0.63(0.54,0.72)
Low-middle SDI	Zambia	177.92(146.54,213.50)	193.35(152.83,239.21)	0.16(0.08,0.25)	169.95(120.65,224.67)	191.05(135.41,253.21)	0.45(0.37,0.52)
Low-middle SDI	Lesotho	178.23(145.37,215.39)	255.95(190.60,329.43)	1.88(1.55,2.21)	173.26(122.40,227.99)	216.99(156.21,278.27)	0.83(0.81,0.85)
Low-middle SDI	Kenya	141.06(119.44,165.44)	159.48(132.81,188.92)	0.61(0.46,0.76)	250.94(180.99,326.16)	262.05(190.14,339.84)	-0.03(-0.13,0.06)
Low-middle SDI	Timor-Leste	180.24(145.64,226.37)	232.05(182.53,286.67)	1.01(0.86,1.16)	324.76(231.58,420.07)	367.55(265.84,470.58)	0.58(0.49,0.67)
Low-middle SDI	Nigeria	169.38(128.85,220.19)	162.61(128.83,197.89)	0.00(-0.15,0.15)	187.20(134.58,247.62)	267.60(196.20,348.92)	1.75(1.57,1.92)
Low-middle SDI	Sudan	378.35(308.70,454.67)	317.98(259.92,391.57)	-0.64(-0.67,-0.61)	292.08(212.36,378.11)	372.73(270.97,482.51)	0.90(0.87,0.93)
Low-middle SDI	Nicaragua	131.29(111.87,150.23)	177.06(142.44,208.31)	0.80(0.46,1.13)	200.60(142.17,263.69)	251.50(178.54,333.19)	0.68(0.60,0.77)
Low-middle SDI	Myanmar	290.75(232.45,358.98)	213.19(177.26,250.06)	-1.22(-1.34,-1.10)	279.51(201.47,360.38)	292.19(209.12,377.26)	0.28(0.20,0.37)

Low-middle SDI	Cabo Verde	137.40(117.40,159.32)	171.02(142.07,198.30)	0.12(-0.18,0.42)	237.78(169.90,314.37)	274.73(198.29,363.00)	0.53(0.48,0.58)
Low-middle SDI	Guatemala	117.87(95.26,143.97)	117.20(91.16,146.94)	-0.01(-0.34,0.33)	127.41(91.50,172.02)	220.49(159.91,290.32)	2.15(2.04,2.27)
Low-middle SDI	Kiribati	285.85(225.71,353.53)	270.97(209.52,339.85)	-0.39(-0.47,-0.31)	343.62(242.80,456.75)	369.25(262.84,481.62)	0.08(-0.02,0.18)
Low-middle SDI	Tajikistan	242.87(207.11,281.54)	398.93(315.64,489.43)	1.86(1.61,2.11)	220.79(159.56,291.17)	221.90(159.99,291.03)	0.02(-0.09,0.13)
Low-middle SDI	Marshall Islands	260.94(206.82,318.25)	279.15(213.08,358.94)	0.29(0.16,0.41)	298.63(210.45,397.45)	353.50(249.14,463.50)	0.47(0.38,0.56)
Low-middle SDI	Morocco	339.09(290.15,384.19)	307.72(240.01,359.26)	-0.50(-0.65,-0.36)	337.94(241.90,437.64)	377.22(272.55,488.50)	0.35(0.26,0.43)
Low-middle SDI	Ghana	198.24(162.76,236.15)	207.34(171.86,248.08)	0.39(0.30,0.48)	232.51(168.37,302.82)	270.31(194.46,349.56)	0.51(0.45,0.57)
Low-middle SDI	Democratic People's Republic of Korea	198.57(156.26,249.63)	170.75(135.43,209.08)	-0.53(-0.75,-0.31)	357.34(251.74,462.37)	285.82(198.56,377.42)	-0.97(-1.07,-0.87)
Low-middle SDI	Maldives	280.34(241.71,319.56)	131.68(104.77,159.94)	-3.24(-3.47,-3.00)	343.88(245.25,444.69)	298.80(216.21,389.73)	-0.60(-0.64,-0.56)
Low-middle SDI	Bolivia (Plurinational State of)	130.58(101.95,164.42)	123.08(92.03,157.12)	-0.21(-0.40,-0.01)	93.32(63.79,128.24)	115.05(81.58,152.59)	0.72(0.69,0.75)
Low-middle SDI	India	176.92(150.29,207.37)	144.48(119.44,169.76)	-0.77(-0.91,-0.64)	167.44(121.02,218.01)	178.29(128.04,230.61)	0.40(0.28,0.52)
Low-middle SDI	Congo	320.95(256.98,387.17)	250.41(200.82,310.05)	-1.00(-1.11,-0.89)	272.60(196.86,354.08)	258.56(188.72,334.47)	-0.26(-0.28,-0.23)
Low-middle SDI	El Salvador	118.67(100.11,137.12)	117.75(86.75,152.01)	0.05(-0.09,0.18)	161.15(115.64,215.51)	222.32(158.41,297.35)	1.60(1.27,1.93)
Low-middle SDI	Eswatini	221.96(179.51,262.58)	243.75(180.16,316.74)	0.63(0.20,1.06)	227.00(162.45,294.33)	265.68(192.21,345.60)	0.58(0.54,0.61)

Low-middle SDI	Micronesia (Federated States of)	277.97(216.95,344.56)	286.60(211.00,364.50)	0.05(0.02,0.08)	318.54(223.06,418.87)	344.43(241.28,452.53)	0.19(0.12,0.26)
Low-middle SDI	Palestine	277.42(216.76,343.37)	211.60(174.54,252.94)	-1.16(-1.29,-1.04)	261.62(188.44,338.78)	280.16(202.38,365.45)	0.11(0.04,0.19)
Low-middle SDI	Tuvalu	243.48(189.13,304.95)	268.68(206.40,344.82)	0.29(0.18,0.41)	269.07(192.35,364.50)	366.90(261.00,485.57)	0.93(0.78,1.09)
Low-middle SDI	Dominican Republic	140.44(115.89,166.30)	177.42(136.19,228.97)	1.71(1.41,2.01)	163.89(116.89,216.63)	210.11(149.71,275.12)	0.89(0.86,0.93)
Low-middle SDI	Kyrgyzstan	221.14(181.18,257.48)	209.80(162.82,262.09)	-0.31(-0.76,0.14)	261.08(184.09,342.41)	185.92(129.57,247.20)	-1.40(-1.49,-1.32)
Low-middle SDI	Belize	139.22(116.47,161.83)	118.93(99.58,139.64)	-0.81(-1.21,-0.41)	175.59(123.96,233.02)	224.16(162.08,295.24)	0.79(0.71,0.87)
Low-middle SDI	Mongolia	405.51(330.41,489.21)	343.87(267.83,427.15)	-1.05(-1.51,-0.59)	336.41(240.77,438.70)	316.35(230.25,412.71)	-0.29(-0.40,-0.18)
Low-middle SDI	Venezuela (Bolivarian Republic of)	197.47(174.75,219.20)	155.24(117.34,201.10)	-1.26(-1.43,-1.10)	221.11(158.82,287.63)	219.38(156.04,285.77)	-0.24(-0.36,-0.13)
Middle SDI	Namibia	231.16(189.88,274.37)	198.86(156.50,247.97)	-0.81(-1.11,-0.50)	238.85(175.40,308.28)	224.98(162.78,288.41)	-0.32(-0.36,-0.27)
Middle SDI	Viet Nam	212.35(174.56,255.46)	197.39(159.79,233.18)	0.09(-0.06,0.23)	279.83(197.93,364.13)	335.17(239.25,436.20)	0.90(0.76,1.05)
Middle SDI	Guyana	379.31(321.07,443.04)	265.14(206.90,332.64)	-1.11(-1.28,-0.93)	213.76(153.05,278.01)	240.42(175.13,311.78)	0.40(0.36,0.44)
Middle SDI	Nauru	396.13(320.44,483.62)	421.85(343.77,507.34)	0.13(-0.25,0.52)	440.74(315.30,569.48)	519.91(379.68,668.01)	0.43(0.31,0.54)
Middle SDI	Syrian Arab Republic	309.98(239.36,379.49)	265.64(195.69,340.18)	-1.01(-1.23,-0.80)	280.65(199.19,370.75)	291.73(210.19,385.30)	0.11(0.08,0.15)
Middle SDI	Philippines	139.66(119.24,162.11)	186.93(152.22,225.29)	1.63(1.23,2.02)	300.95(213.39,386.05)	306.81(221.09,401.54)	-0.04(-0.09,0.00)

Middle SDI	Tokelau	190.68(148.75,241.24)	205.73(160.91,259.30)	0.20(0.03,0.37)	270.63(192.40,365.47)	376.67(273.26,485.24)	1.05(0.91,1.19)
Middle SDI	Saint Vincent and the Grenadines	208.55(176.98,240.68)	173.46(144.78,202.98)	-0.58(-0.88,-0.28)	193.06(137.63,257.21)	235.85(169.15,310.50)	0.69(0.63,0.76)
Middle SDI	Uzbekistan	239.39(198.32,280.66)	490.07(379.33,598.54)	2.50(1.81,3.19)	249.79(177.53,328.61)	295.46(212.28,387.03)	0.63(0.58,0.68)
Middle SDI	Colombia	144.46(122.99,165.29)	73.53(54.55,95.52)	-2.55(-2.70,-2.41)	164.89(118.65,214.74)	163.53(115.33,214.42)	0.01(-0.05,0.06)
Middle SDI	Botswana	228.36(180.70,287.00)	227.01(170.15,296.89)	-0.28(-0.61,0.04)	243.48(176.17,317.06)	271.12(199.11,344.62)	0.37(0.35,0.39)
Middle SDI	Suriname	184.29(156.55,212.27)	144.87(116.03,176.63)	-0.96(-1.28,-0.63)	182.82(130.63,243.27)	214.30(153.98,279.59)	0.52(0.49,0.56)
Middle SDI	Tonga	127.78(104.43,154.16)	150.65(121.15,184.37)	0.59(0.39,0.79)	321.51(228.81,423.76)	406.29(290.00,525.24)	0.81(0.75,0.86)
Middle SDI	Paraguay	139.89(117.14,162.42)	123.80(93.93,157.28)	-0.37(-0.51,-0.23)	191.54(140.61,251.00)	209.04(150.34,272.60)	0.22(0.19,0.26)
Middle SDI	Brazil	197.31(174.65,218.93)	104.78(91.22,116.25)	-2.20(-2.26,-2.15)	221.15(158.66,286.72)	199.58(144.71,259.48)	-0.42(-0.46,-0.39)
Middle SDI	Ecuador	88.84(72.73,105.36)	98.04(75.21,124.90)	0.96(0.61,1.32)	103.91(73.10,138.63)	143.63(101.76,191.20)	1.23(1.15,1.31)
Middle SDI	Samoa	240.48(197.96,290.38)	235.43(187.81,291.64)	-0.14(-0.21,-0.07)	363.17(258.43,469.00)	406.46(291.14,536.02)	0.27(0.14,0.40)
Middle SDI	Peru	74.29(58.27,92.83)	58.17(42.36,76.78)	-0.46(-0.83,-0.08)	89.29(62.00,120.68)	139.65(99.96,182.86)	1.83(1.63,2.04)
Middle SDI	Mexico	112.19(95.23,128.73)	118.40(97.73,142.22)	0.32(0.24,0.40)	205.57(146.01,271.07)	254.92(181.99,330.60)	0.82(0.75,0.89)
Middle SDI	Algeria	393.00(315.83,472.60)	235.45(185.57,286.97)	-1.92(-2.03,-1.81)	303.94(219.71,395.14)	298.00(212.62,390.26)	-0.31(-0.41,-0.20)
Middle SDI	Gabon	242.64(199.69,288.	212.93(169.96,253.	-0.52(-0.66,-0.	224.20(163.15,294.	239.39(173.13,313.	0.17(0.13,0.21

		88)	31)	39)	50)	00)	)
Middle SDI	Egypt	309.71(262.26,358.72)	314.94(238.39,403.48)	0.43(0.26,0.59)	247.61(176.57,331.57)	355.62(257.64,463.08)	1.62(1.46,1.78)
Middle SDI	Indonesia	241.72(210.90,273.07)	273.62(230.75,309.40)	0.64(0.54,0.73)	422.13(306.93,541.68)	482.98(350.62,618.50)	0.51(0.48,0.54)
Middle SDI	Fiji	274.92(223.38,330.34)	283.35(226.64,351.45)	-0.12(-0.28,0.03)	370.52(267.79,482.61)	472.46(341.33,608.28)	0.84(0.8,0.89)
Middle SDI	Cuba	142.73(117.84,169.09)	96.98(75.05,122.12)	-1.58(-1.78,-1.38)	164.54(118.74,216.32)	161.76(116.76,214.95)	-0.16(-0.24,-0.09)
Middle SDI	Grenada	228.08(194.28,263.31)	163.70(139.57,186.84)	-1.09(-1.30,-0.88)	200.45(142.48,264.43)	242.29(173.55,318.06)	0.61(0.56,0.65)
Middle SDI	Iran (Islamic Republic of)	244.79(209.54,279.57)	157.77(135.35,179.10)	-1.76(-1.89,-1.63)	258.40(185.43,336.87)	262.87(190.07,342.26)	0.03(-0.03,0.09)
Middle SDI	Saint Lucia	246.51(218.31,275.64)	135.36(110.56,160.98)	-2.27(-2.71,-1.84)	243.39(175.11,315.24)	242.39(173.93,315.06)	0.09(0.03,0.16)
Middle SDI	Turkmenistan	328.19(273.30,382.74)	336.26(260.16,418.35)	-0.52(-0.88,-0.16)	294.45(209.21,380.90)	338.25(241.47,436.89)	0.58(0.53,0.64)
Middle SDI	Iraq	331.99(275.15,396.07)	296.57(238.44,350.01)	-0.59(-0.67,-0.51)	376.42(270.84,489.21)	416.48(302.83,535.36)	0.41(0.38,0.44)
Middle SDI	Tunisia	210.75(173.41,251.81)	175.52(128.07,227.82)	-0.81(-0.91,-0.71)	217.01(155.47,286.05)	269.12(194.00,350.94)	0.59(0.50,0.68)
Middle SDI	South Africa	155.23(136.17,174.62)	161.79(143.26,179.15)	0.10(-0.44,0.63)	283.71(204.86,367.32)	281.66(206.25,360.88)	-0.12(-0.18,-0.06)
Middle SDI	Costa Rica	133.33(113.17,151.30)	93.67(70.54,118.75)	-1.66(-1.87,-1.45)	246.75(177.83,320.25)	257.94(184.80,334.65)	0.10(0.05,0.15)
Middle SDI	Albania	245.36(210.07,282.75)	183.21(135.01,239.76)	-0.81(-1.00,-0.62)	320.00(228.53,412.75)	290.41(204.92,374.90)	-0.23(-0.31,-0.15)
Middle SDI	Azerbaijan	281.62(240.08,327.47)	376.58(303.97,449.04)	1.06(0.86,1.26)	268.33(188.74,355.11)	273.63(194.28,356.24)	0.17(0.03,0.31)

Middle SDI	Jamaica	144.76(125.98,163.37)	120.88(93.77,151.48)	-0.36(-0.71,-0.01)	166.57(119.29,221.93)	227.67(163.39,298.45)	1.26(1.09,1.44)
Middle SDI	Equatorial Guinea	272.21(201.82,348.74)	192.33(144.83,247.05)	-1.50(-1.72,-1.28)	226.87(164.13,293.33)	255.33(186.85,329.74)	0.46(0.39,0.53)
Middle SDI	China	190.99(159.60,226.22)	153.34(126.19,182.49)	-0.47(-0.60,-0.35)	253.57(180.16,339.44)	323.55(230.58,421.54)	1.03(0.98,1.09)
Middle SDI	Panama	116.30(96.78,134.37)	82.18(61.33,105.28)	-1.16(-1.29,-1.04)	181.77(130.59,238.30)	214.04(153.69,277.38)	0.50(0.46,0.55)
Middle SDI	Thailand	109.07(88.98,130.35)	70.00(50.49,92.44)	-1.85(-1.99,-1.71)	250.36(177.22,333.06)	240.70(171.70,317.66)	-0.10(-0.17,-0.03)
Middle SDI	Armenia	260.85(216.53,305.49)	197.93(156.40,240.00)	-1.56(-1.76,-1.36)	281.51(203.66,363.77)	252.72(179.99,329.22)	-0.34(-0.36,-0.32)
High-middle SDI	Sri Lanka	197.35(167.85,228.26)	133.83(100.51,174.21)	-1.11(-1.35,-0.87)	295.45(211.54,381.85)	311.00(220.65,400.65)	0.31(0.22,0.40)
High-middle SDI	Republic of Moldova	344.17(285.75,405.04)	258.10(216.69,304.73)	-1.29(-1.57,-1.02)	273.27(196.64,353.08)	284.53(202.72,370.06)	0.18(0.10,0.26)
High-middle SDI	Uruguay	152.10(126.41,177.23)	90.27(76.21,103.17)	-1.95(-2.07,-1.84)	189.55(135.37,251.98)	170.14(121.52,223.45)	-0.50(-0.61,-0.40)
High-middle SDI	Georgia	430.62(362.54,496.39)	290.14(236.61,340.12)	-1.46(-1.66,-1.26)	355.07(257.39,455.97)	310.61(223.20,401.09)	-0.54(-0.62,-0.47)
High-middle SDI	Mauritius	292.18(259.89,323.37)	167.25(133.12,203.67)	-2.53(-2.84,-2.22)	399.24(288.58,512.54)	362.11(256.83,472.29)	-0.66(-0.88,-0.45)
High-middle SDI	Argentina	143.04(117.63,171.13)	106.80(91.50,121.21)	-0.85(-0.94,-0.77)	140.72(99.71,189.14)	163.98(118.74,213.44)	0.90(0.71,1.09)
High-middle SDI	Lebanon	259.43(209.18,317.50)	191.32(138.58,228.38)	-0.79(-0.99,-0.59)	257.73(183.51,338.93)	314.38(225.75,410.65)	0.88(0.73,1.02)
High-middle SDI	Libya	200.50(160.45,243.25)	188.73(146.12,232.31)	-0.15(-0.31,0.00)	291.05(207.53,380.69)	357.70(260.46,465.02)	0.78(0.70,0.86)
High-middle SDI	Niue	251.21(203.11,301.	248.01(194.31,299.	-0.22(-0.37,-0.	365.46(262.87,483.	447.60(320.42,577.	0.65(0.54,0.76

		49)	60)	08)	46)	04)	)
High-middle SDI	American Samoa	184.27(158.39,215.63)	199.05(163.85,236.13)	0.13(0.03,0.22)	368.25(261.38,480.24)	447.92(327.66,575.21)	0.58(0.45,0.72)
High-middle SDI	Bosnia and Herzegovina	258.42(219.36,295.38)	191.69(144.92,245.46)	-1.29(-1.48,-1.10)	350.75(251.26,456.42)	342.16(240.23,450.00)	0.05(-0.01,0.11)
High-middle SDI	Kazakhstan	331.12(285.52,373.96)	310.12(260.57,360.48)	-0.82(-1.42,-0.21)	372.00(265.02,479.05)	328.74(236.03,422.26)	-0.49(-0.57,-0.41)
High-middle SDI	Seychelles	254.23(225.58,282.19)	181.39(156.81,208.07)	-1.39(-1.48,-1.29)	386.22(279.10,500.15)	373.41(265.79,485.05)	-0.21(-0.24,-0.18)
High-middle SDI	Dominica	205.02(176.51,236.01)	168.85(137.02,204.93)	-0.51(-0.68,-0.34)	202.25(144.85,263.28)	231.10(165.85,305.43)	0.45(0.38,0.52)
High-middle SDI	Jordan	276.37(231.66,323.60)	168.49(137.79,199.52)	-2.15(-2.37,-1.93)	356.19(257.04,465.59)	349.57(248.77,459.31)	-0.13(-0.21,-0.04)
High-middle SDI	Ukraine	301.62(248.03,348.82)	313.67(251.32,383.57)	-0.52(-0.85,-0.19)	301.35(211.15,392.02)	274.60(196.76,362.37)	-0.34(-0.43,-0.25)
High-middle SDI	Malaysia	217.67(190.96,241.99)	174.12(137.08,214.92)	-0.91(-1.05,-0.78)	387.71(277.61,499.41)	412.15(292.22,523.06)	0.55(0.35,0.75)
High-middle SDI	Palau	196.67(149.65,253.34)	227.75(177.18,287.36)	0.43(0.30,0.56)	307.52(218.85,401.08)	432.57(306.46,571.46)	1.08(0.86,1.30)
High-middle SDI	Barbados	143.13(122.03,163.75)	113.24(92.18,135.90)	-0.99(-1.10,-0.88)	191.74(138.64,252.30)	232.49(167.35,309.38)	0.69(0.63,0.75)
High-middle SDI	Antigua and Barbuda	173.25(148.87,198.62)	144.00(120.80,168.59)	-0.77(-0.98,-0.57)	203.57(144.56,263.47)	231.92(166.90,304.52)	0.46(0.43,0.50)
High-middle SDI	Portugal	186.95(154.77,219.35)	68.51(57.35,79.42)	-3.90(-4.10,-3.70)	253.90(182.65,334.20)	156.17(111.51,204.20)	-1.72(-1.77,-1.67)
High-middle SDI	North Macedonia	374.83(326.55,421.57)	329.68(262.63,405.82)	-0.52(-0.76,-0.27)	385.98(275.79,502.67)	339.96(242.90,441.54)	-0.38(-0.45,-0.31)
High-middle SDI	Belarus	280.82(229.96,323.17)	272.23(205.76,341.02)	-0.45(-0.85,-0.05)	297.42(211.87,386.21)	301.04(213.14,390.08)	0.03(-0.03,0.10)

High-middle SDI	Saint Kitts and Nevis	330.79(281.34,379.05)	188.01(156.59,221.45)	-1.89(-2.13,-1.65)	244.43(174.53,318.17)	254.06(182.65,329.14)	0.08(0.01,0.15)
High-middle SDI	Turkey	227.16(189.37,261.90)	133.60(104.79,165.29)	-1.83(-1.97,-1.70)	269.21(194.02,347.77)	263.08(190.23,341.88)	-0.12(-0.42,0.19)
High-middle SDI	Bahrain	332.42(273.24,394.55)	147.77(115.60,184.64)	-2.98(-3.25,-2.72)	280.84(203.66,364.46)	276.65(201.61,363.09)	-0.08(-0.11,-0.04)
High-middle SDI	Trinidad and Tobago	245.12(213.89,275.43)	149.94(112.43,194.94)	-2.23(-2.47,-1.99)	216.57(156.89,283.79)	258.36(186.57,337.42)	0.60(0.51,0.68)
High-middle SDI	Chile	162.51(140.40,182.53)	91.54(79.42,101.78)	-1.84(-1.93,-1.74)	205.59(146.43,266.86)	182.33(131.70,236.84)	-0.47(-0.51,-0.44)
High-middle SDI	Romania	394.17(343.13,438.22)	238.82(191.42,287.70)	-2.26(-2.50,-2.02)	416.34(300.41,535.92)	340.40(244.54,438.11)	-0.81(-0.86,-0.77)
High-middle SDI	Greenland	208.58(172.72,248.53)	105.10(82.06,129.12)	-2.93(-3.19,-2.68)	303.91(217.20,404.24)	222.73(159.59,296.85)	-1.10(-1.22,-0.98)
High-middle SDI	Bulgaria	389.88(329.67,448.68)	322.98(253.36,398.39)	-1.17(-1.44,-0.90)	370.58(264.13,476.53)	333.43(236.95,431.40)	-0.32(-0.41,-0.22)
High-middle SDI	Cook Islands	221.86(186.04,255.78)	174.85(145.08,207.51)	-0.84(-0.98,-0.71)	315.88(226.18,411.63)	419.91(300.20,541.10)	0.88(0.78,0.99)
High-middle SDI	Serbia	366.98(318.23,415.34)	271.97(216.70,335.43)	-1.28(-1.50,-1.06)	402.15(289.97,519.76)	333.42(240.87,427.83)	-0.73(-0.80,-0.66)
High-middle SDI	Spain	140.32(119.05,160.34)	59.25(49.17,67.63)	-3.12(-3.23,-3.02)	212.32(152.03,276.42)	147.08(106.01,193.33)	-1.38(-1.44,-1.32)
High-middle SDI	Northern Mariana Islands	177.63(144.13,215.72)	183.47(152.22,217.58)	0.15(0.07,0.24)	358.81(252.93,473.21)	423.86(301.00,553.13)	0.42(0.22,0.61)
High-middle SDI	Oman	287.49(215.20,371.19)	274.45(225.06,326.83)	0.47(0.24,0.71)	214.42(153.48,281.63)	335.06(242.62,432.76)	2.05(1.83,2.28)
High-middle SDI	Hungary	367.85(326.61,400.65)	167.76(131.82,208.42)	-2.98(-3.15,-2.80)	453.76(327.13,583.61)	262.89(188.09,345.73)	-2.16(-2.26,-2.05)
High-middle SDI	Montenegro	253.79(213.74,294.	249.63(201.56,300.	0.15(-0.01,0.3	346.97(250.08,449.	311.19(222.54,402.	-0.30(-0.36,-0.

		90)	19)	1)	53)	19)	23)
High-middle SDI	Croatia	304.57(258.00,347.92)	158.91(120.09,197.50)	-2.14(-2.25,-2.03)	349.67(246.70,455.52)	263.53(187.02,342.24)	-0.91(-0.98,-0.84)
High-middle SDI	Greece	192.10(164.94,217.83)	98.63(81.97,114.07)	-2.79(-3.02,-2.55)	227.39(162.57,294.31)	156.92(112.16,206.73)	-1.41(-1.51,-1.31)
High-middle SDI	Bahamas	196.96(171.10,220.74)	164.03(132.88,199.16)	-0.65(-0.76,-0.54)	203.79(147.66,272.37)	226.54(161.85,297.23)	0.40(0.35,0.45)
High-middle SDI	United States Virgin Islands	187.84(153.23,223.68)	177.59(145.14,206.66)	0.15(-0.04,0.33)	179.02(128.11,238.32)	212.77(153.06,275.99)	0.64(0.55,0.72)
High-middle SDI	Italy	161.25(139.77,180.58)	66.13(54.31,77.05)	-3.40(-3.55,-3.26)	223.83(159.88,289.30)	156.89(112.19,207.36)	-1.24(-1.37,-1.10)
High-middle SDI	Malta	193.28(160.43,223.05)	83.94(67.34,101.10)	-2.90(-2.99,-2.81)	225.38(161.85,293.34)	182.46(132.64,240.71)	-0.75(-0.84,-0.66)
High-middle SDI	Poland	261.39(220.89,303.13)	123.35(99.21,149.97)	-2.86(-3.01,-2.71)	306.12(217.79,396.09)	258.39(184.91,335.73)	-0.38(-0.58,-0.19)
High-middle SDI	Israel	171.44(148.02,193.51)	64.59(53.22,73.83)	-3.82(-4.06,-3.57)	203.87(146.20,265.63)	166.39(119.28,218.02)	-0.74(-0.79,-0.69)
High-middle SDI	Russian Federation	317.19(274.28,357.31)	239.47(197.27,279.52)	-1.50(-2.02,-0.98)	341.58(248.55,437.51)	306.63(221.90,392.60)	-0.39(-0.43,-0.35)
High-middle SDI	Saudi Arabia	233.19(184.00,287.93)	209.61(167.63,250.73)	-0.37(-0.62,-0.13)	264.44(189.77,347.98)	338.21(241.70,441.48)	0.83(0.64,1.02)
High SDI	Slovakia	322.00(272.14,367.55)	176.95(131.60,222.97)	-2.05(-2.20,-1.90)	379.57(268.51,489.78)	309.29(221.33,404.80)	-0.61(-0.66,-0.56)
High SDI	Bermuda	191.89(159.25,225.46)	79.43(62.65,98.85)	-3.14(-3.41,-2.86)	176.56(126.28,235.32)	180.32(128.96,238.28)	0.01(-0.02,0.04)
High SDI	Guam	213.60(180.81,248.58)	164.10(133.17,197.99)	-1.10(-1.46,-0.74)	320.76(229.97,416.84)	444.42(316.90,575.88)	1.09(0.94,1.25)
High SDI	Puerto Rico	136.97(114.85,158.33)	74.66(56.13,95.71)	-2.26(-2.40,-2.12)	199.34(142.88,259.86)	231.42(167.01,303.10)	0.68(0.56,0.81)

High SDI	Latvia	326.25(275.59,377.40)	210.91(168.45,254.75)	-1.88(-2.15,-1.62)	324.36(230.76,422.03)	306.87(218.50,395.08)	-0.20(-0.23,-0.16)
High SDI	Brunei Darussalam	273.99(236.00,313.42)	154.60(128.40,182.40)	-1.95(-2.08,-1.82)	338.80(246.18,439.61)	223.89(159.80,291.29)	-1.63(-1.76,-1.5)
High SDI	Czechia	286.19(240.97,333.41)	113.16(83.24,144.45)	-3.22(-3.37,-3.07)	367.06(264.24,477.71)	258.01(181.19,340.40)	-0.91(-1.08,-0.75)
High SDI	Qatar	310.20(245.23,372.16)	206.71(155.46,261.91)	-1.53(-1.81,-1.25)	279.88(203.56,358.73)	282.47(204.66,365.57)	-0.20(-0.40,0.00)
High SDI	France	106.27(91.32,119.91)	49.45(40.10,57.69)	-2.90(-3.04,-2.76)	212.48(152.76,275.65)	161.28(116.13,213.74)	-1.04(-1.17,-0.90)
High SDI	Estonia	319.25(265.86,372.69)	179.52(136.75,223.57)	-2.32(-2.48,-2.16)	319.29(228.77,412.45)	250.39(179.00,331.44)	-0.91(-0.99,-0.82)
High SDI	Australia	155.62(131.24,177.02)	53.82(43.37,64.42)	-4.19(-4.47,-3.90)	229.08(164.09,298.54)	164.99(117.70,219.61)	-1.25(-1.34,-1.16)
High SDI	New Zealand	172.56(148.91,194.41)	69.19(55.59,81.38)	-3.48(-3.67,-3.30)	209.81(148.57,273.89)	148.30(104.04,194.16)	-1.18(-1.27,-1.08)
High SDI	Slovenia	211.58(160.63,266.62)	89.81(67.64,114.68)	-3.20(-3.32,-3.09)	335.01(239.02,440.25)	248.29(178.60,324.82)	-1.06(-1.13,-0.99)
High SDI	Cyprus	239.42(198.21,278.97)	112.30(91.96,133.67)	-3.11(-3.26,-2.96)	194.01(139.24,253.05)	163.68(115.41,215.85)	-0.74(-0.87,-0.62)
High SDI	Lithuania	279.48(231.92,325.19)	195.93(152.92,242.07)	-1.32(-1.55,-1.08)	318.59(230.40,411.97)	298.22(213.45,388.44)	-0.30(-0.45,-0.15)
High SDI	United Kingdom	200.38(175.74,220.69)	62.61(52.42,72.13)	-4.76(-5.11,-4.41)	238.48(170.89,307.93)	145.36(104.08,191.45)	-2.02(-2.19,-1.86)
High SDI	Austria	170.10(140.76,198.97)	90.39(74.25,104.64)	-2.42(-2.54,-2.29)	252.32(180.54,329.91)	208.14(146.82,272.65)	-0.73(-0.80,-0.66)
High SDI	Belgium	123.17(100.75,145.60)	66.80(55.13,76.39)	-2.29(-2.39,-2.19)	181.54(128.92,238.23)	180.14(130.79,236.69)	0.04(-0.02,0.10)
High SDI	Kuwait	182.77(156.19,206.	115.25(92.91,138.6	-1.46(-1.91,-1.	268.24(193.81,354.	281.58(203.14,367.	0.30(0.13,0.47

		30)	6)	01)	51)	39)	)
High SDI	Finland	227.90(199.07,254.45)	95.63(77.37,111.43)	-3.04(-3.25,-2.83)	266.98(193.64,343.63)	188.24(134.66,248.55)	-1.17(-1.22,-1.11)
High SDI	United States of America	151.01(127.83,171.56)	81.64(68.57,94.21)	-2.56(-2.80,-2.32)	276.49(199.27,356.81)	240.25(173.74,311.13)	-0.48(-0.57,-0.39)
High SDI	Singapore	148.02(126.69,169.49)	47.26(38.08,55.77)	-4.39(-4.62,-4.15)	269.47(191.53,351.78)	141.06(99.69,185.14)	-2.80(-3.04,-2.56)
High SDI	Ireland	209.88(180.89,237.14)	74.29(60.22,86.56)	-4.17(-4.43,-3.90)	226.73(161.56,297.13)	161.13(115.77,210.60)	-1.72(-1.96,-1.47)
High SDI	Taiwan (Province of China)	154.83(136.66,171.64)	63.71(49.44,80.74)	-3.11(-3.39,-2.83)	331.35(239.99,426.38)	315.95(222.68,408.66)	-0.22(-0.34,-0.11)
High SDI	Iceland	122.47(99.72,145.92)	57.06(45.07,68.86)	-2.98(-3.11,-2.85)	163.47(117.93,215.96)	145.66(104.84,191.71)	-0.33(-0.37,-0.29)
High SDI	Japan	118.05(101.21,131.90)	42.01(34.09,48.61)	-3.74(-3.94,-3.54)	267.19(193.22,341.07)	215.61(153.59,279.56)	-0.87(-0.98,-0.76)
High SDI	Sweden	154.29(129.85,178.97)	75.56(61.55,88.46)	-2.66(-2.75,-2.57)	254.97(179.33,332.18)	233.57(164.97,308.67)	-0.22(-0.25,-0.18)
High SDI	Canada	125.05(103.77,145.35)	54.26(42.62,64.50)	-3.34(-3.59,-3.09)	266.65(190.56,350.51)	180.66(127.73,240.87)	-1.29(-1.52,-1.05)
High SDI	Republic of Korea	213.68(181.67,242.88)	45.15(35.43,55.71)	-6.03(-6.31,-5.74)	300.31(213.73,391.54)	140.81(100.51,184.12)	-3.37(-3.63,-3.11)
High SDI	United Arab Emirates	381.64(317.13,461.82)	215.26(168.17,266.16)	-2.14(-2.59,-1.68)	410.41(296.66,524.04)	374.22(269.49,486.71)	-0.35(-0.44,-0.26)
High SDI	Netherlands	131.39(111.41,149.23)	61.14(50.14,71.03)	-3.43(-3.79,-3.06)	181.83(130.35,234.73)	157.17(111.28,207.14)	-0.81(-0.98,-0.63)
High SDI	San Marino	94.43(75.50,114.60)	64.35(43.78,89.79)	-1.25(-1.40,-1.11)	220.02(156.33,293.96)	182.80(131.28,243.51)	-0.72(-0.79,-0.66)
High SDI	Denmark	191.63(164.19,215.27)	67.82(56.12,78.76)	-4.16(-4.37,-3.95)	232.05(166.76,298.08)	162.14(115.00,212.04)	-1.38(-1.46,-1.30)

High SDI	Andorra	95.16(73.88,126.98)	62.94(47.14,80.09)	-1.50(-1.71,-1.29)	196.03(139.06,258.57)	160.83(116.37,210.97)	-0.88(-0.96,-0.79)
High SDI	Luxembourg	178.94(150.50,207.06)	67.00(53.56,79.72)	-3.58(-3.66,-3.49)	242.13(173.96,318.65)	194.58(140.36,254.37)	-0.82(-0.96,-0.68)
High SDI	Germany	216.39(185.14,243.84)	97.82(81.72,112.18)	-2.95(-3.30,-2.60)	262.34(189.23,338.57)	190.70(136.71,250.33)	-1.29(-1.46,-1.11)
High SDI	Monaco	134.31(101.37,166.08)	74.93(56.37,91.42)	-2.07(-2.29,-1.85)	202.00(140.94,265.52)	171.26(123.63,227.89)	-0.67(-0.74,-0.60)
High SDI	Norway	175.33(152.72,196.24)	60.23(48.97,70.12)	-4.07(-4.23,-3.92)	242.65(174.64,312.26)	199.90(143.01,258.94)	-0.62(-0.70,-0.54)
High SDI	Switzerland	112.41(92.44,131.88)	55.77(43.85,66.81)	-2.57(-2.66,-2.49)	167.14(119.32,218.81)	117.76(82.81,156.26)	-1.11(-1.29,-0.92)

ASMRs, age-standardized mortality rates; ASYRs, age-standardized rates of years lived with disability; EAPC, estimated annual percentage change; UI, uncertainty interval; CI, confidence interval.

Table S5 21 GBD regions and their country compositions.

GBD regions	Countries
**Central Asia**	Armenia, Azerbaijan, Georgia, Kazakhstan, Kyrgyzstan, Mongolia, Tajikistan, Turkmenistan, Uzbekistan
**Central Europe**	Albania, Bosnia and Herzegovina, Bulgaria, Croatia, Czech Republic, Hungary, Montenegro, North Macedonia, Poland, Romania, Serbia, Slovakia, Slovenia
**Eastern Europe**	Belarus, Estonia, Latvia, Lithuania, Moldova, Russia, Ukraine
**Australasia**	Australia, New Zealand
**High-income Asia Pacific**	Brunei, Japan, South Korea, Singapore
**High-income North America**	Canada, Greenland, USA
**Southern Latin America**	Argentina, Chile, Uruguay
**Western Europe**	Andorra, Austria, Belgium, Sweden, Switzerland, UK, Cyprus, Denmark, Finland, France, Germany, Greece, Iceland, Ireland, Israel, Italy, Luxembourg, Malta, Monaco, Netherlands, Norway, Portugal, San Marino, Spain
**Andean Latin America**	Bolivia, Ecuador, Peru
**Caribbean**	Antigua and Barbuda, Grenada, Guyana, Haiti, Jamaica, Puerto Rico, Saint Kitts and Nevis, The Bahamas, Barbados, Belize, Bermuda, Cuba, Dominica, Dominican Republic, Saint Lucia, Saint Vincent and the Grenadines, Suriname, Trinidad and Tobago, Virgin Islands
**Central Latin America**	Colombia, Costa Rica, Venezuela, Panama, Honduras, El Salvador, Guatemala, Mexico, Nicaragua
**Tropical Latin America**	Brazil, Paraguay
**North Africa and Middle East**	Afghanistan, Algeria, Bahrain, Egypt, Iran, Iraq, Jordan, Kuwait, Lebanon, Libya, Morocco, Oman, Palestine, Qatar, Saudi Arabia, Sudan, Syria, Tunisia, Turkey, United Arab Emirates, Yemen
**South Asia**	Bangladesh, Bhutan, India, Nepal, Pakistan
**East Asia**	China, North Korea, Taiwan (province of China)
**Oceania**	American Samoa, Northern Mariana Islands, Palau, Papua New Guinea, Samoa, Solomon Islands, Tokelau, Tonga, Tuvalu, Vanuatu, Cook Islands, Fiji, Guam, Kiribati, Marshall Islands, Federated States of Micronesia, Nauru, Niue
**Southeast Asia**	Cambodia, Thailand, Timor-Leste, Vietnam, Sri Lanka, Indonesia, Lao People's Democratic Republic, Malaysia, Maldives, Mauritius, Myanmar, Philippines, Seychelles
**Central sub-Saharan Africa**	Angola, Central African Republic, Congo (Brazzaville), DR Congo, Equatorial Guinea, Gabon
**Eastern sub-Saharan Africa**	Burundi, Comoros, Djibouti, Eritrea, Ethiopia, Kenya, Madagascar, Malawi, Mozambique, Rwanda, Somalia, South Sudan, Uganda, Tanzania, Zambia
**Southern sub-Saharan Africa**	Botswana, Eswatini, Lesotho, Namibia, South Africa, Zimbabwe
**Western sub-Saharan Africa**	Benin, Burkina Faso, Cape Verde, Cameroon, Chad, Côte d'Ivoire, The Gambia, Ghana, Guinea-Bissau, Guinea, Liberia, Mali, Mauritania, Niger, Nigeria, São Tomé and PrÍncipe, Senegal, Sierra Leone, Togo

Figure S1

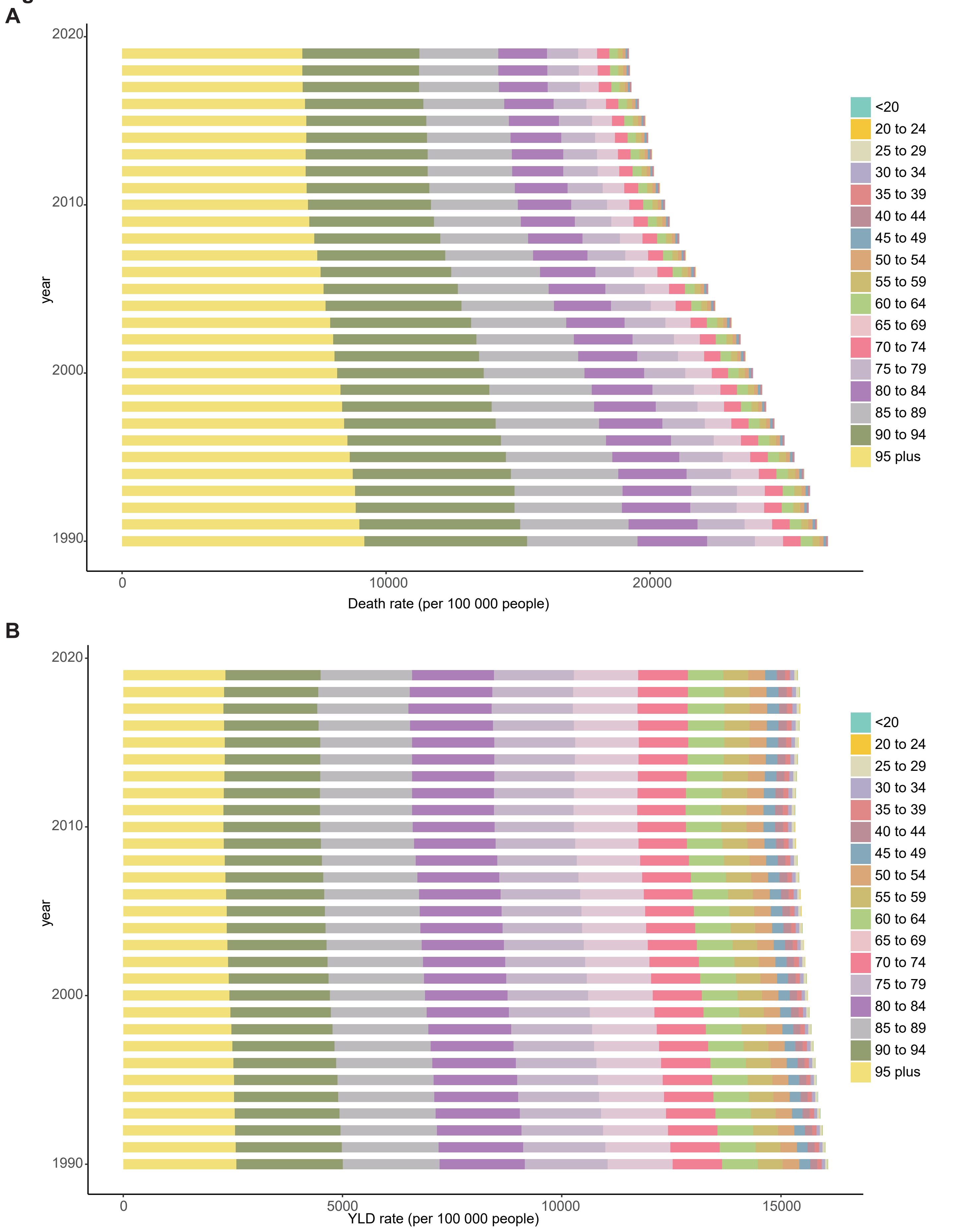

Figure S1. Age-specific rate of deaths and YLDs attributable to HSBP in 1990-2019. (A) Age-specific rate of deaths attributable to HSBP in 1990-2019. (B) Age-specific rate of YLDs attributable to HSBP in 1990-2019. YLDs = Years lived with disability; HSBP = high systolic blood pressure.

Figure S2

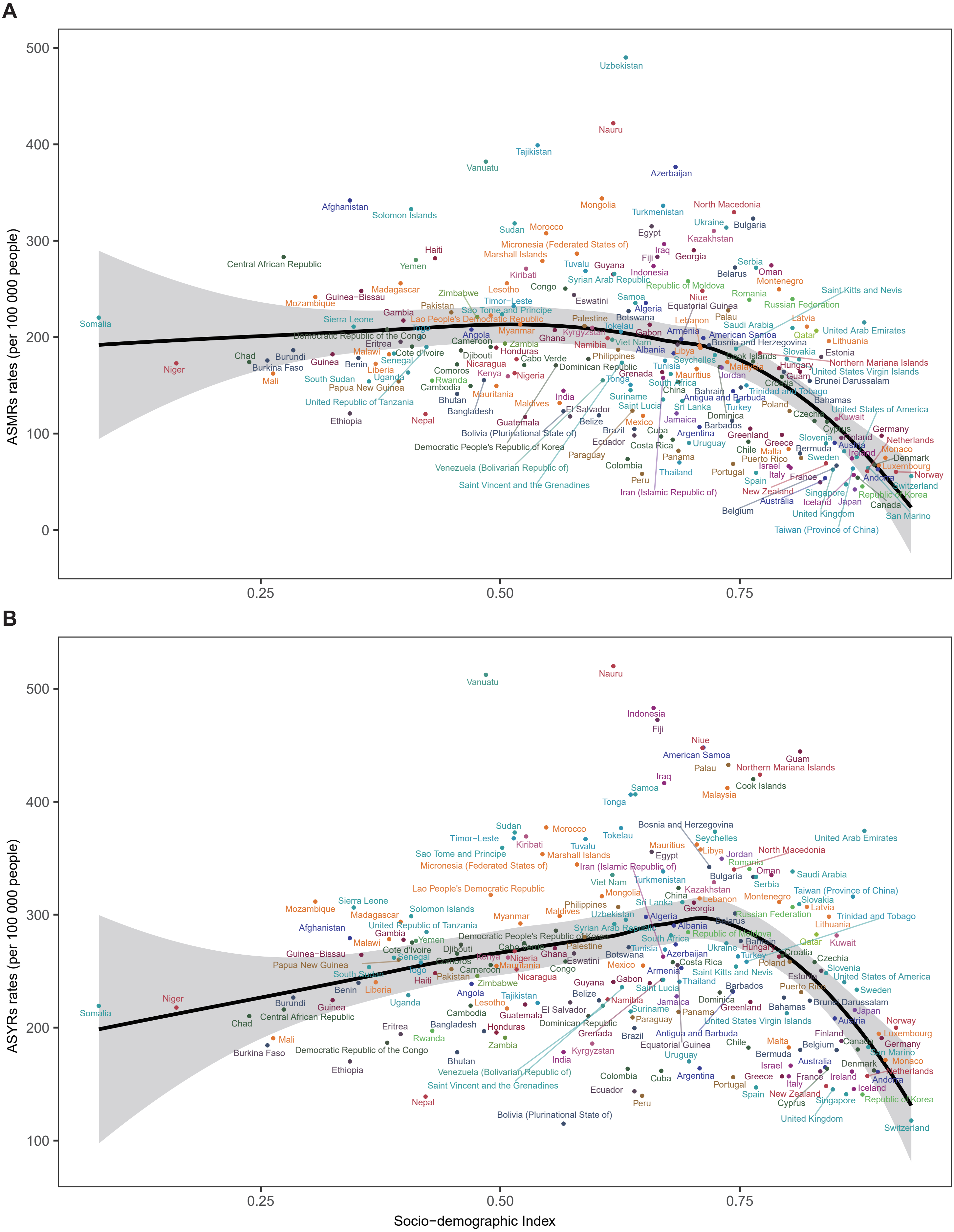

Figure S2 ASMRs and ASYRs attributable to HSBP across 204 countries and territories by socio-demographic index for both sexes combined in 2019. (A) ASMRs. (B) ASYRs. ASMRs = age-standardized mortality rates; ASYRs = age-standardized rates of years lived with disability; HSBP = high systolic blood pressure.

Figure S3

Region	NRHD Male	NRHD Female	RHD Male	RHD Female	PAD Male	PAD Female	CM Male	CM Female	AFF Male	AFF Female	AA Male	AA Female
Eastern Sub−Saharan Africa	0.23	0.19	0.23	0.24	0.26	0.25	0.34	0.30	0.33	0.34	0.37	0.34
East Asia	0.19	0.16	0.24	0.26	0.24	0.25	0.12	0.12	0.31	0.33	0.33	0.34
Western Sub−Saharan Africa	0.25	0.20	0.28	0.29	0.29	0.32	0.36	0.37	0.36	0.39	0.40	0.41
Oceania	0.18	0.14	0.17	0.17	0.18	0.20	0.22	0.21	0.24	0.27	0.27	0.29
Central Sub−Saharan Africa	0.24	0.20	0.26	0.28	0.27	0.28	0.36	0.35	0.35	0.38	0.38	0.38
Central Latin America	0.28	0.22	0.27	0.25	0.24	0.24	0.28	0.28	0.33	0.33	0.35	0.32
Global	0.25	0.23	0.24	0.25	0.25	0.25	0.23	0.26	0.32	0.34	0.34	0.33
Caribbean	0.24	0.20	0.25	0.27	0.23	0.22	0.18	0.26	0.31	0.31	0.33	0.31
South Asia	0.22	0.19	0.24	0.25	0.24	0.25	0.13	0.15	0.31	0.34	0.33	0.34
Central Asia	0.23	0.16	0.31	0.28	0.27	0.28	0.37	0.37	0.36	0.39	0.39	0.38
Andean Latin America	0.21	0.17	0.19	0.19	0.20	0.22	0.23	0.22	0.27	0.30	0.27	0.27
Tropical Latin America	0.28	0.23	0.28	0.25	0.27	0.26	0.32	0.32	0.34	0.34	0.38	0.35
Central Europe	0.27	0.22	0.31	0.28	0.30	0.26	0.26	0.30	0.37	0.36	0.41	0.37
Southern Sub−Saharan Africa	0.28	0.26	0.26	0.29	0.30	0.31	0.38	0.39	0.36	0.40	0.38	0.40
Southeast Asia	0.26	0.23	0.25	0.23	0.29	0.29	0.32	0.33	0.35	0.38	0.36	0.36
North Africa and Middle East	0.22	0.17	0.25	0.27	0.24	0.26	0.27	0.25	0.31	0.35	0.35	0.36
Eastern Europe	0.32	0.27	0.34	0.32	0.30	0.29	0.12	0.20	0.37	0.39	0.42	0.41
Southern Latin America	0.29	0.27	0.24	0.23	0.28	0.27	0.30	0.29	0.35	0.34	0.34	0.34
Australasia	0.24	0.23	0.24	0.24	0.22	0.23	0.17	0.24	0.30	0.31	0.31	0.31
High−income Asia Pacific	0.25	0.21	0.24	0.22	0.24	0.21	0.28	0.24	0.33	0.30	0.34	0.29
High−income North America	0.23	0.24	0.21	0.22	0.20	0.23	0.20	0.25	0.28	0.32	0.28	0.30
Western Europe	0.28	0.26	0.26	0.26	0.26	0.26	0.23	0.27	0.34	0.34	0.35	0.33

Region	Endocarditis Male	Endocarditis Female	OCCD Male	OCCD Female	Stroke Male	Stroke Female	IHD Male	IHD Female	CKD Male	CKD Female	HHD Male	HHD Female
Eastern Sub−Saharan Africa	0.37	0.33	0.38	0.36	0.52	0.52	0.53	0.51	0.63	0.63	1.00	1.00
East Asia	0.31	0.30	0.30	0.30	0.49	0.49	0.50	0.51	0.65	0.66	1.00	1.00
Western Sub−Saharan Africa	0.36	0.34	0.43	0.43	0.57	0.59	0.57	0.60	0.69	0.70	1.00	1.00
Oceania	0.26	0.27	0.28	0.32	0.39	0.43	0.41	0.44	0.57	0.60	1.00	1.00
Central Sub−Saharan Africa	0.39	0.37	0.40	0.42	0.54	0.56	0.55	0.56	0.66	0.67	1.00	1.00
Central Latin America	0.34	0.30	0.34	0.31	0.51	0.49	0.52	0.50	0.60	0.59	1.00	1.00
Andean Latin America	0.26	0.24	0.27	0.26	0.41	0.42	0.43	0.45	0.56	0.56	1.00	1.00
Global	0.33	0.31	0.35	0.33	0.51	0.52	0.52	0.53	0.62	0.62	1.00	1.00
Caribbean	0.34	0.31	0.34	0.35	0.50	0.50	0.51	0.50	0.58	0.57	1.00	1.00
South Asia	0.33	0.33	0.35	0.36	0.50	0.54	0.51	0.54	0.57	0.58	1.00	1.00
Central Asia	0.40	0.37	0.43	0.41	0.56	0.56	0.54	0.56	0.54	0.51	1.00	1.00
Tropical Latin America	0.37	0.33	0.38	0.34	0.52	0.50	0.55	0.53	0.62	0.61	1.00	1.00
Central Europe	0.43	0.36	0.45	0.39	0.55	0.52	0.57	0.54	0.67	0.63	1.00	1.00
Southern Sub−Saharan Africa	0.41	0.41	0.42	0.43	0.56	0.58	0.58	0.61	0.70	0.72	1.00	1.00
Southeast Asia	0.36	0.33	0.38	0.38	0.57	0.59	0.56	0.58	0.66	0.67	1.00	1.00
North Africa and Middle East	0.33	0.35	0.34	0.36	0.49	0.52	0.52	0.55	0.66	0.66	1.00	1.00
Eastern Europe	0.47	0.40	0.46	0.44	0.55	0.53	0.58	0.57	0.63	0.61	1.00	1.00
Southern Latin America	0.35	0.31	0.35	0.33	0.50	0.49	0.51	0.50	0.66	0.66	1.00	1.00
Australasia	0.32	0.31	0.29	0.24	0.44	0.44	0.49	0.48	0.61	0.61	1.00	1.00
High−income Asia Pacific	0.32	0.27	0.33	0.27	0.48	0.42	0.50	0.45	0.65	0.63	1.00	1.00
High−income North America	0.28	0.29	0.25	0.20	0.42	0.44	0.44	0.47	0.64	0.64	1.00	1.00
Western Europe	0.35	0.32	0.36	0.32	0.48	0.47	0.53	0.52	0.68	0.67	1.00	1.00

0.8 0.6 0.4 0.2 0

Figure S3. Fraction of disease ASMRs attributable to HSBP by GBD region for female and male in 2019. ASMRs = age-standardized mortality rates; HSBP = high systolic blood pressure; GBD = global burden of disease,injuries, and risk factors study; IHD, ischemic heart disease; NRHD = non−rheumatic valvular heart disease; OCCD = other cardiovascular and circulatory diseases; PAD = peripheral artery disease; RHD = rheumatic heart disease; AA = aortic aneurysm; AFF = atrial fibrillation and flutter; CM = cardiomyopathy and myocarditis; CKD = chronic kidney disease; HHD = hypertensive heart disease.

Figure S4

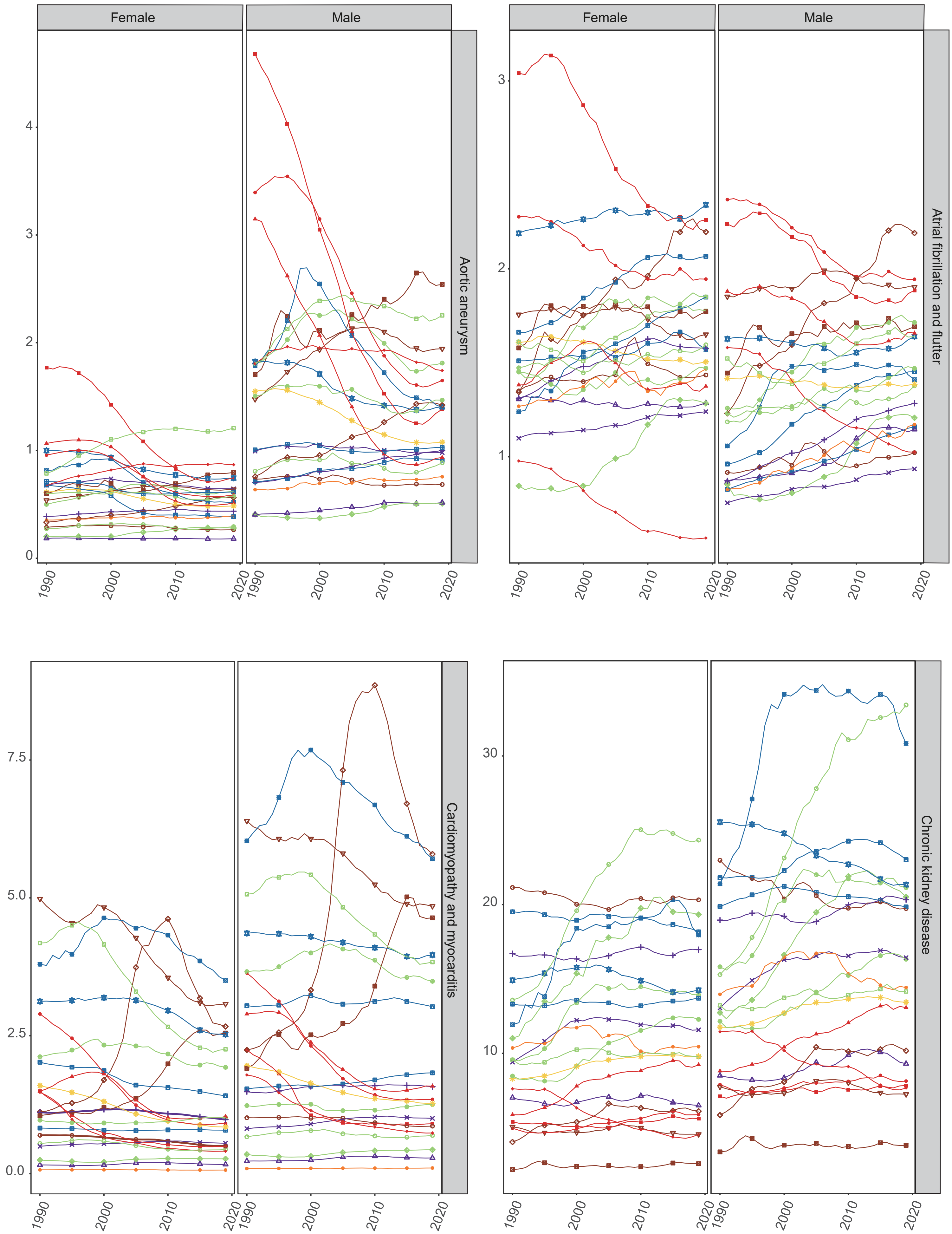

Female
Male
Aortic aneurysm
Atrial fibrillation and flutter
Cardiomyopathy and myocarditis
Chronic kidney disease
0
1
2
3
4
7.5
5.0
2.5
0.0
10
20
30
1990
2000
2010
2020

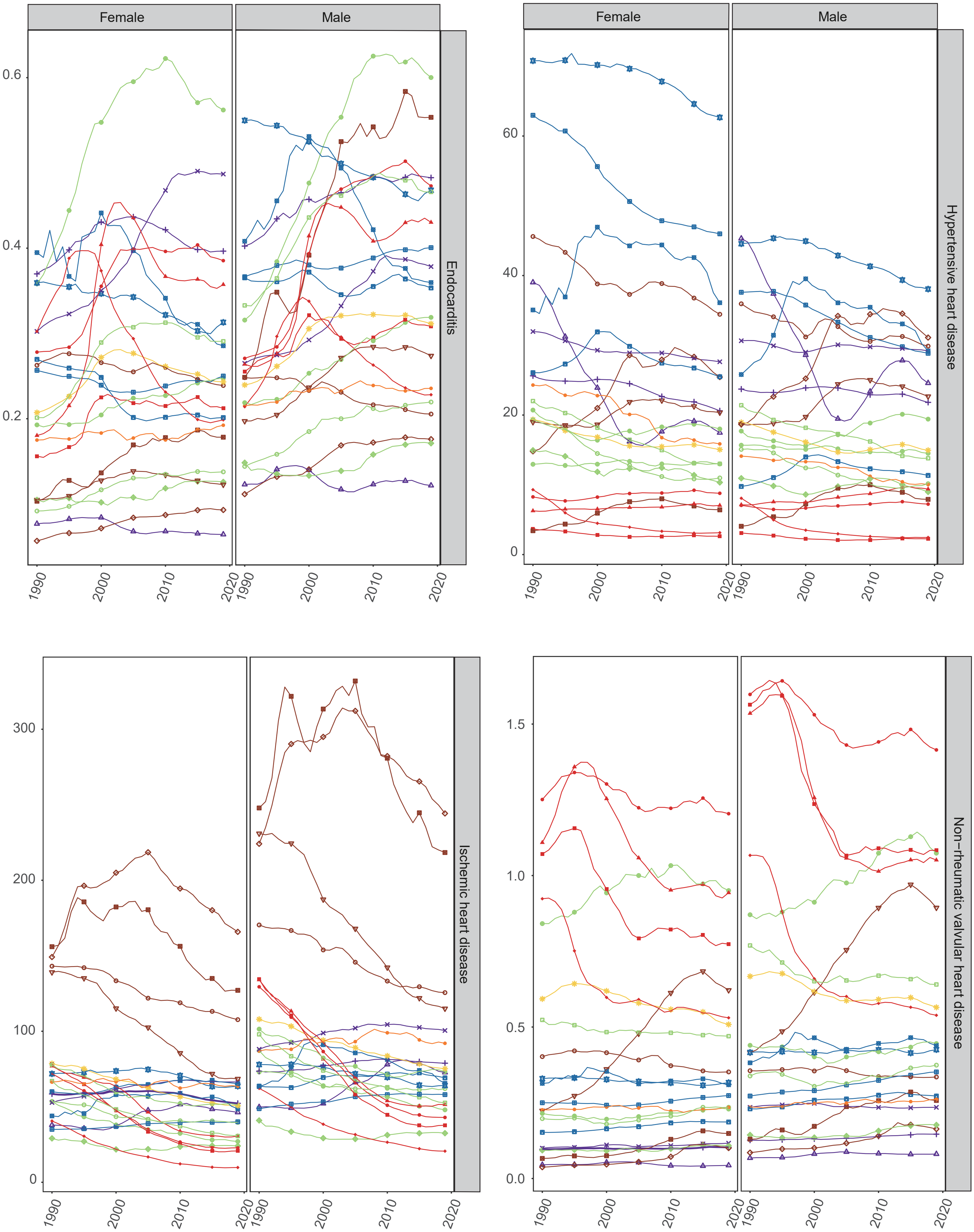

Female
Male
Endocarditis
0.2
0.4
0.6
Female
Male
Hypertensive heart disease
0
20
40
60
Ischemic heart disease
0
100
200
300
Non-rheumatic valvular heart disease
0.0
0.5
1.0
1.5
1990
2000
2010
2020

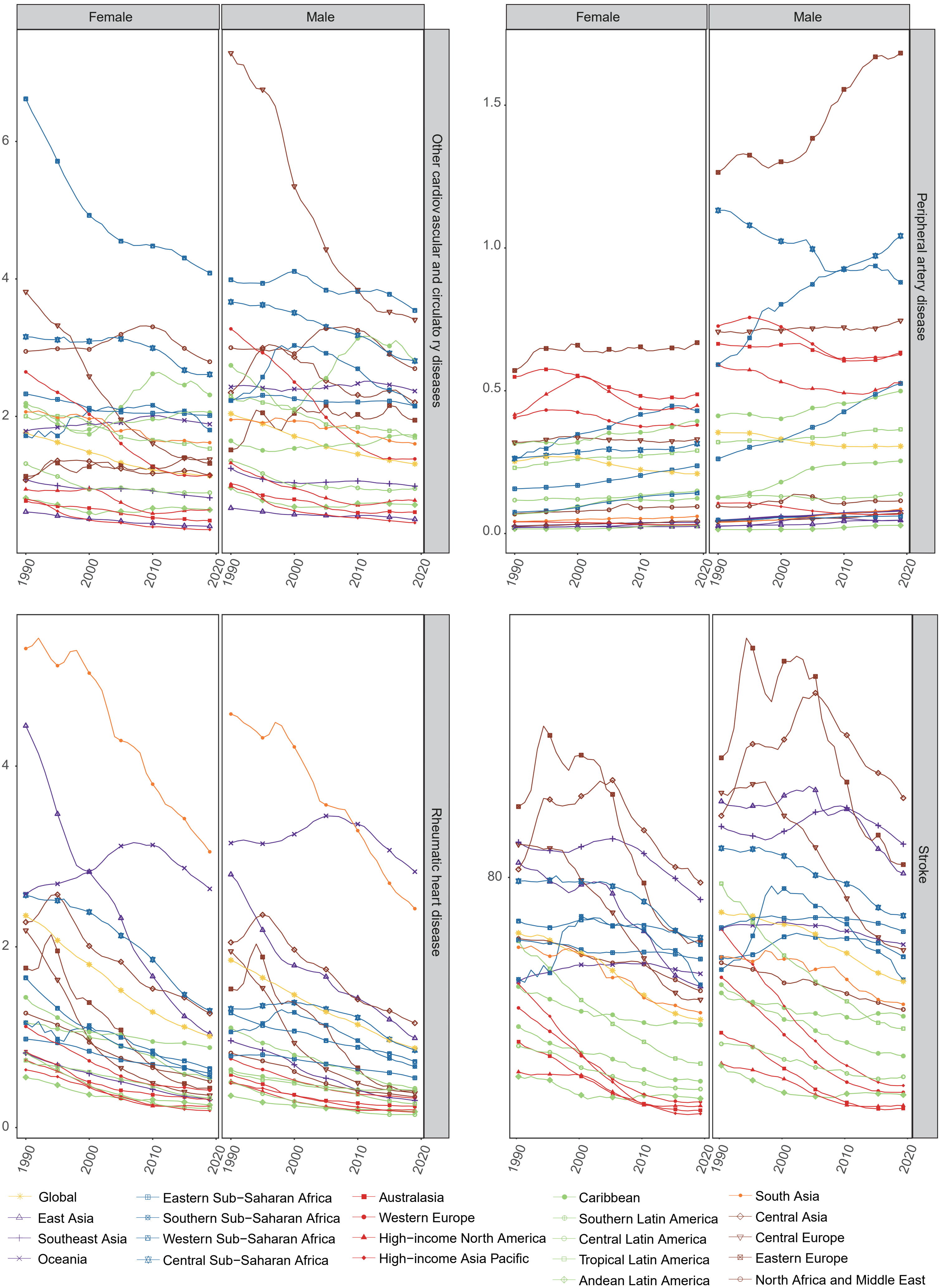

Figure S4. ASMRs of 12 causes attributable to HSBP across GBD regions for female and male , 1990–2019. Abbreviations as in **Figure S3**.

Figure S5

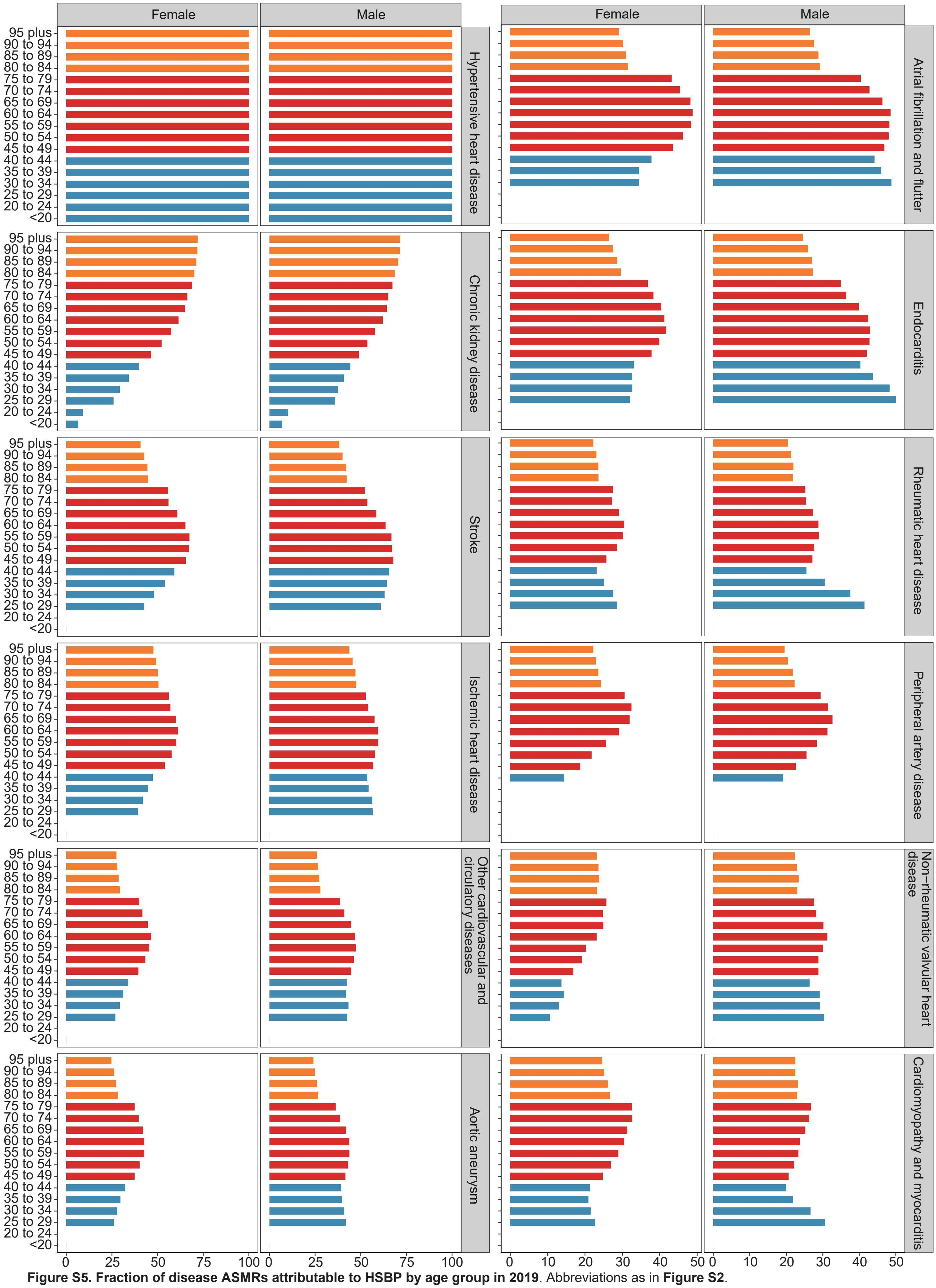

Figure S5. Fraction of disease ASMRs attributable to HSBP by age group in 2019. Abbreviations as in **Figure S2**.

Figure S6

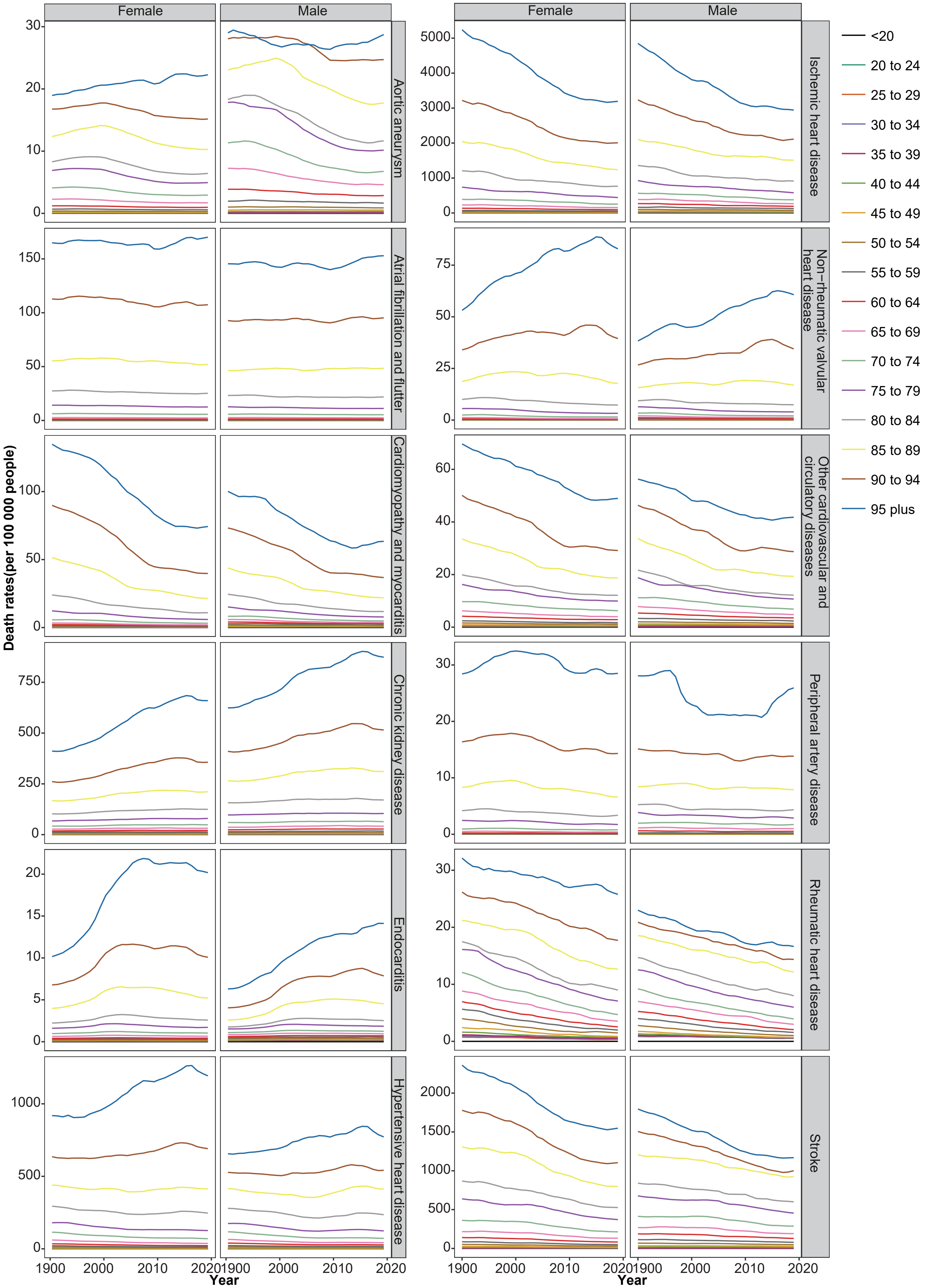

Figure S6 ASMRs of 12 causes attributable to HSBP for female and male by age, 1990–2019. Abbreviations as in **Figure S2**.
